# Comparative genomic analysis of *Acinetobacter* spp. plasmids originating from clinical settings and environmental habitats

**DOI:** 10.1038/s41598-018-26180-3

**Published:** 2018-05-17

**Authors:** Ileana P. Salto, Gonzalo Torres Tejerizo, Daniel Wibberg, Alfred Pühler, Andreas Schlüter, Mariano Pistorio

**Affiliations:** 1 IBBM (Instituto de Biotecnología y Biología Molecular), CCT-CONICET-La Plata, Departamento de Ciencias Biológicas, Facultad de Ciencias Exactas, Universidad Nacional de La Plata, Calles 47 y 115 (1900), La Plata, Argentina; 2 Center for Biotechnology (CeBiTec), Bielefeld University, Genome Research of Industrial Microorganisms, Universitätsstr. 27, D-33615 Bielefeld, Germany

## Abstract

Bacteria belonging to the genus *Acinetobacter* have become of clinical importance over the last decade due to the development of a multi-resistant phenotype and their ability to survive under multiple environmental conditions. The development of these traits among *Acinetobacter* strains occurs frequently as a result of plasmid-mediated horizontal gene transfer. In this work, plasmids from nosocomial and environmental *Acinetobacter* spp. collections were separately sequenced and characterized. Assembly of the sequenced data resulted in 19 complete replicons in the nosocomial collection and 77 plasmid contigs in the environmental collection. Comparative genomic analysis showed that many of them had conserved backbones. Plasmid coding sequences corresponding to plasmid specific functions were bioinformatically and functionally analyzed. Replication initiation protein analysis revealed the predominance of the Rep_3 superfamily. The phylogenetic tree constructed from all *Acinetobacter* Rep_3 superfamily plasmids showed 16 intermingled clades originating from nosocomial and environmental habitats. Phylogenetic analysis of relaxase proteins revealed the presence of a new sub-clade named MOBQ_Aci_, composed exclusively of *Acinetobacter* relaxases. Functional analysis of proteins belonging to this group showed that they behaved differently when mobilized using helper plasmids belonging to different incompatibility groups.

## Introduction

The genetic and functional diversity of bacteria facilitates their adaptation to different environments. Bacterial genome rearrangement involves gene loss, gene duplication and gene acquisition through **H**orizontal **G**ene **T**ransfer (HGT)^1^. Frequently, acquisition of new genes is mediated by the introduction of mobile genetic elements (MGEs) like plasmids, integrative conjugative elements, transposons and bacteriophages. Plasmid transfer is often indicated as the most efficient DNA exchange among prokaryotes^2^. Hence, MGEs, particularly plasmids, constitute a gene reservoir available among different bacterial species^3^ and are important for the dissemination of genes for adaptation, evolution and interactions with plant or animal hosts^4–6^.

Plasmids are selfish genetic elements that undergo autonomous and self-controlled replication^7^. These molecules represent assemblies of different functional modules such as replication, mobilization and maintenance, as well as accessory genes  that may provide adaptive advantages to host bacteria, such as pathogenicity/virulence determinants, xenobiotic compound degradation or antimicrobial resistance mechanisms^8^. Moreover, as plasmids can be found in every branch of the phylogenetic tree, an integrative view of plasmid ecology is needed to understand community adaptation. Different environmental settings have distinct bacterial community compositions, which determine the type of dominant plasmids that can be found^9^. When the plasmid-encoded information involves resistance mechanisms, virulence factors or other adaptive and advantageous functions, recipient bacteria become more competitive compared with plasmid-free ancestors.

Antibiotic multi-resistant phenotypes have had a significant impact over the last decade since they have appeared in bacteria associated with nosocomial infections in hospitals and intensive care units (ICUs)^10–12^. When these genes enter MGEs such as plasmids, information spreads rapidly among bacterial communities, giving rise to multi-resistant bacteria epidemics^13–16^. Since plasmids are able to self-replicate, transfer, persist and even acquire new genes within their backbones, they can be considered as specialized HGT vehicles, and have therefore become an important target of research to design new strategies for the control of antimicrobial resistance dissemination^17,18^.

Bacteria belonging to the genus *Acinetobacter* have gained clinical relevance due to the emergence of multi-resistant strains and as one of the most important agents responsible for colonization and severe infections in ICU patients^14,19–21^. The ability of *Acinetobacter* spp. to grow under a great variety of environmental conditions such as low nutrient, broad pH range and highly selective pressure habitats has been associated with bacterial genomic plasticity^22^. New gene acquisition is frequently mediated by HGT mechanisms, where plasmids play a very important role; thus, study of their biology may provide new molecular evidence to understand the importance of HGT in bacterial adaptation to hospital environments. Although several studies have described plasmids carrying either antimicrobial resistance genes or xenobiotic degradation operons^23–36^, the biology of *Acinetobacter* plasmids remains largely unexplored.

In this study, the plasmid content of two *Acinetobacter* spp. collections originating from nosocomial and environmental habitats was studied in order to further characterize *Acinetobacter* spp. plasmids and to investigate genetic differences between specific plasmid modules of strains isolated from clinical and environmental sources. After isolation, high-throughput sequencing and annotation, plasmid sequences were thoroughly analyzed, and their predicted replication, maintenance and mobilization systems as well as adaptive traits were investigated.

## Results

### Plasmid content of *Acinetobacter* spp. isolates recovered from two different environmental habitats of Argentina

Plasmid content was analyzed in two bacteria collections. The nosocomial collection consisted of 64 multi-resistant *Acinetobacter* spp. isolates from five different hospitals, and the environmental collection consisted of 59 *Acinetobacter* spp. bacteria isolated from La Plata city ground soil and water samples. The analysis of plasmid content in both bacterial collections was performed with Eckhardt-type gels^37,38^. Plasmid profiles revealed that 85 to 89% of isolates from the nosocomial and environmental collections harbored at least one plasmid of quite variable size (not shown). Further characterization was achieved by plasmid DNA purification and high-throughput sequencing on the Illumina MiSeq system.

### High-throughput sequencing of plasmid DNAs

Sequencing of nosocomial plasmid collection samples yielded 1,419,341 sequence reads, amounting to 347,374,930 bp and reaching an approximate 650-fold coverage value. After assembly of the sequence reads, 40 scaffolds could be identified; of these, 23 corresponded to 19 completely closed replicons. Closed plasmid sizes varied from 1.9 to 62 kb, consistent with the plasmid profiles obtained in Eckhard-type gels. Replicon GC content ranged from 32.9 to 60.8% (Table 1). Considering the average chromosomal GC content of *Acinetobacter* spp. reported in the literature (*ca*. 40%), differences were significant in eight replicons, suggesting that they might have been recently acquired by HGT.

**Table 1:** General characteristics of the nosocomial plasmid collection.

Replicon	Size (bp)	% GC content	CDS N°	CDS with predicted functions			
				Replication	Mobilization	Maintenance	Resistance
pIH1	61669	50.83	68	3	3	8	20
pIH2	42507	49.49	57	1	16	4	—
pIH3	49725	41.84	43	1	—	1	—
phIH4	14125	36.73	15	—	—	—	—
phIH5	9501	39.31	12	—	—	—	—
pIH6	6481	40.38	9	1	4	—	—
pIH7	5273	34.99	6	1	2	—	1
pIH8	9156	34.81	14	1	1	2	—
pIH9	10229	34.14	17	2	1	—	-
phIH10	6699	37.78	12	1	—	—	—
pIH11	7349	33.02	9	2	—	2	—
pIH12	6780	37.79	12	—	3	—	—
pIH13	4135	42.42	4	—	2	—	1
pIH14	5629	37.29	12	1	—	—	—
pIH15	5351	60.77	9	—	—	—	—
pIH16	8752	36.76	11	1	2	2	—
pIH17	1846	39.54	2	1	—	—	—
pIH18	3979	32.92	6	2	—	—	—
pIH19	2177	39.27	2	1	—	—	—

Environmental plasmid collection samples amounted to 1,340,077 reads, covering a total of 384,534,478 bp and reaching an approximate 186-fold coverage value. After assembly, 846 contigs were obtained and arranged in 158 scaffolds. In this case, it was not possible to complete any circular replicon *in silico*. Thus, only 77 contigs harboring coding sequences (CDS) homologous to specific plasmid functions (replication, maintenance, conjugation and/or mobilization) were selected for further analyses (Table S1).

All sequences were then automatically annotated using the GenDB^39^ platform and manually curated. *In silico* functional classification allowed the identification of genes with specific plasmid functions in both collections. Plasmid sequences from the nosocomial collection comprised a total of 320 CDS; 23% encoded proteins with specific plasmid functions, 4% hypothetical proteins and 73% other functions, such as DNA binding/modification/restriction/recombination, other MGEs and resistance determinants. Plasmid sequences from the 77 environmental plasmid contigs were predicted to harbor 616 CDS from which 23.9% encoded putative homologous proteins associated with specific plasmid functions, 48.5% hypothetical proteins and 27.6% other protein functions. Functional assignments of the predicted CDS from both collections are shown in Fig. S1.

### Comparative genomic analysis of closed plasmid replicons

Gene content and backbone gene organization of closed replicons obtained after sequence assembly of the hospital plasmid collection were analyzed to determine plasmid homology. The whole molecules were therefore compared with other database sequences to identify similarities and/or differences with other plasmids.

Plasmid pIH1 was the largest closed replicon isolated from the nosocomial collection. *In silico* comparison of this replicon with other plasmids from databases revealed its homology to plasmids pENT-4bd (CP008907.1) and p34998 (CP012169.1) isolated from *Enterobacter cloacae* ECR091 and *Enterobacter hormaechei* 34998, respectively. Homologous regions of database plasmids and pIH1 contained type II toxin-antitoxin (TA) system protein genes (Table S2), three replication initiation proteins (Rep) (Table S3) and a conserved heavy metal-resistance cluster comprising silver/copper resistance genes (not shown).

**Figure 1:**
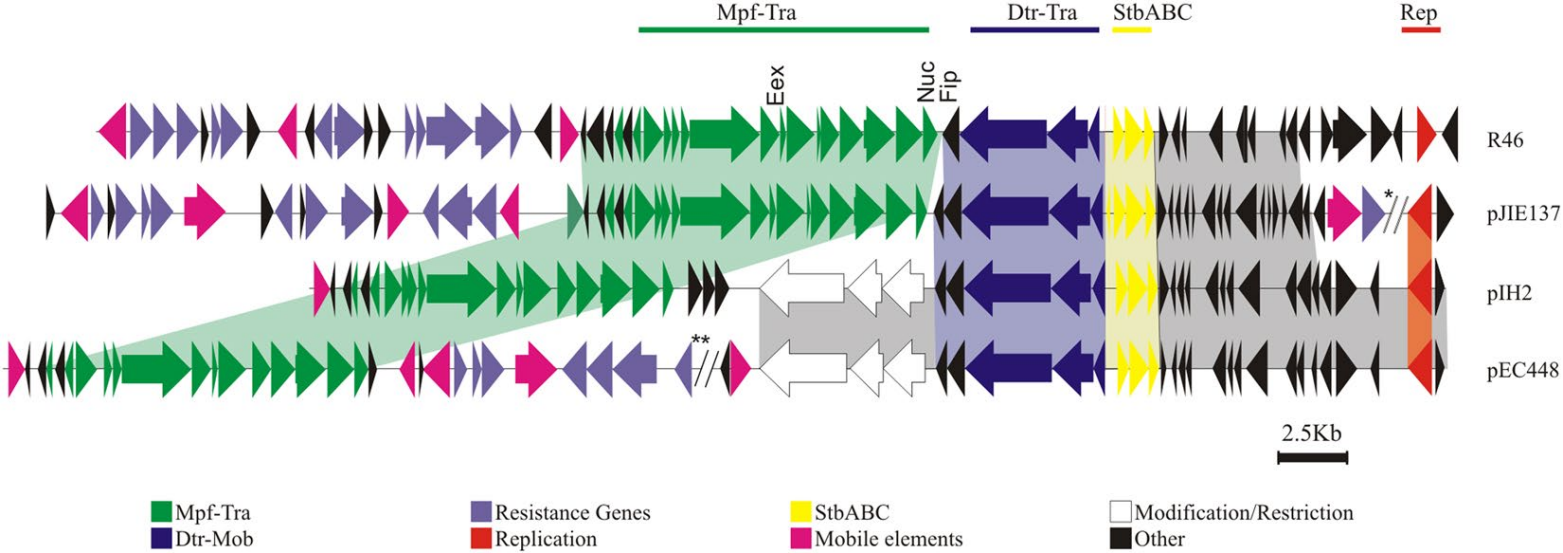
Backbone comparison of pIH2 and IncN plasmids. Alignments of pIH2 to homologous IncN2 plasmids from database pJIE137 (EF219134) (75% coverage, 99% identity). Plasmid pEC448 (CP015078) (91% coverage, 99% identity) and IncN plasmid R46 (AY046276) are shown. Similarities among mobilization (Mpf-green, Dtr-blue), maintenance (StbABC-yelllow) and replication (Rep-red) modules and other regulatory and hypothetical proteins (gray) are highlighted in color. Two main differences arose from backbone comparison: an 11 Kb insertion in pIH2, comprising hypothetical and DNA restriction-modification proteins, and absence of antibiotic resistance genes associated with mobile-transposable elements, as found in the other plasmids. The replication initiation protein found in pIH2, homologous to that found in pEC448 and pJIE137, corresponded to a novel RepA protein. Therefore, these plasmids were classified as IncN2 plasmids. *// and **// indicate fragments of 5.7 and 15.9 Kb respectively, omitted in order to fit the molecules into the graphic.

Nucleotide sequence analysis of pIH2 revealed a high degree of nucleotide identity with plasmids pEC448-OXA63 (CP015078) and pJIE137^40^ isolated from clinical enterobacteria (Fig. 1). Phylogenetic analysis based on the relaxase gene showed that pIH2 belonged to the MOB_F11_ family, according to the plasmid classification scheme developed by Francia *et al*.^41^ and Garcillán-Barcia *et al*.^42^ (see below). Members of this plasmid group include incompatibility (Inc) plasmids IncN, IncW and IncP-9. Further bioinformatics analysis showed that the characteristics harbored by pIH2 backbone were similar to those previously found in IncN2 plasmids. In particular, pIH2 shared a high degree of sequence identity with the complete conjugation, stabilization and Rep modules of IncN2 plasmid pJIE137^40^. In addition, pIH2 backbone showed 99% DNA identity with plasmid pEC448-OXA63 isolated from multi-resistant enterobacteria from a Buenos Aires City hospital. Alignment of pIH2 with pEC448-OXA63 and pJIE137 revealed two major differences. First, plasmids pIH2 and pEC448-OXA63 shared an insertion within the *fip-nuc* integration site of the IncN backbone. This insertion comprised three CDS encoding restriction/modification enzymes flanked by recombinase genes, as well as seven hypothetical genes only found in pIH2. The second and more interesting difference was that pIH2 lacked resistance genes, integrons and other associated transposable elements that are usually present in IncN-type replicons (Fig. 1).

Comparative nucleotide sequence analysis of pIH3 revealed a high degree of similarity with plasmids WZ85p2^43^, p6200-114.848 (CP010398.1) and pORAB01-1(CP015484.1) (Fig. S2), which originate from *Acinetobacter baumanni* strains. Alignment between pIH3 and ZW85p2 showed that a segment of 112,800 bp was absent in the pIH3 backbone, downstream of a CDS encoding a putative phage tail protein. The corresponding ZW85p2 fragment encoded phage proteins, DNA restriction, modification and binding proteins, as well as integrases and transposable elements associated with tetracycline resistance (Fig. S2). Further search in phage databases showed that ZW85p2 encoded for a complete *Salmonella* SSU5 prophage^44^. The same analysis in pIH3 showed that only 13 CDS from the complete replicon were homologous to phage genes from the *Salmonella* SSU5 phage.

Nucleotide analysis of pIH6 revealed no sequence homology to other complete plasmids in databases. Analysis of putative mobilization (Mob) genes showed that they were homologous to those found on an *Acinetobacter junnii* CIP 107470 5.8 kb contig (APPS01000010). Alignment of this contig and pIH6 revealed that the *rep* gene present on pIH6 was missing in the contig. The shared backbone consisted of six CDS: four related to conjugal transfer, one involved in plasmid maintenance and one hypothetical gene (not shown).

Search in databases revealed that pIH7 was homologous to pIP1858^29^ and p1ABAYE^45^ Rep and Mob modules. However, accessory genes were different. In particular, pIH7 carried a putative TetR family transcriptional regulator gene and the ethidium bromide-methyl viologen resistance gene (*emrE*) (Fig. S3).

Analysis of plasmids pIH8, pIH9, pIH11 and pIH18 showed that none of them shared significant nucleotide identity and coverage with other plasmids in databases. Only specific plasmid modules such as putative Rep or TA CDS were homologous to other *A*. *baumannii* plasmids in databases, suggesting the presence of new and undescribed plasmid sequences and architectures in the nosocomial plasmid collection (not shown).

Plasmid pIH12 shared nucleotide sequence similarities to small segments of *Acinetobacter* spp. plasmids in databases, including an IS17 insertion sequence as well as a set of putative hypothetical genes. Similar results were obtained in plasmid pIH15, which showed nucleotide sequence similarity to a segment present in enterobacterial plasmids harboring an IS*Pa*38 insertion sequence as well as other hypothetical genes (not shown).

**Figure 2:**
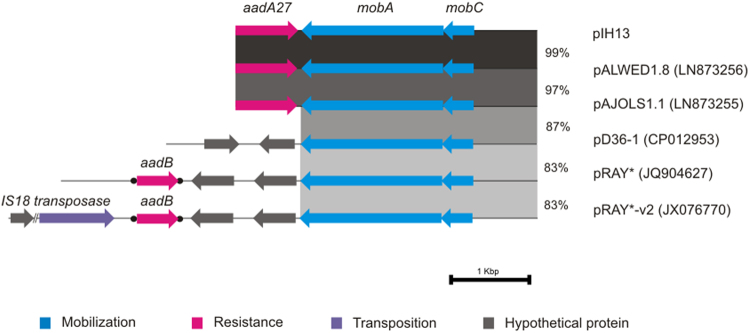
Backbone comparison of pIH13 and related plasmids from database. The alignment of pIH13 with homologous pRAY-like plasmids from database is shown. Plasmid pIH13 from the nosocomial collection was almost identical to plasmids pALWED1.8 (100% coverage, 99% identity) and pAJOLS1.1 (100% coverage, 97% identity) of *Acinetobacter* species isolated from arctic permafrost. The highly conserved backbone of these three plasmids comprised the non-coding region as well as the mobilization module MobAC (light blue) and the aminoglycoside resistance gene *aadA*27 (pink). Comparison of these plasmids with pRAY-like plasmids from database showed high similarities between non-coding regions and the mobilization modules. However, differences were found when the aminoglycoside resistance genes were addressed. Neither plasmids from artic permafrost nor pIH13 harbored the typical aminoglycoside gene cassette commonly found in pRAY plasmid isolates from clinical environments. *Nucleotide percentage of identity is shown and represented in scale of gray. Dot-flanked arrows represent gene cassettes*.

Plasmid pIH13 was found related to pRAY plasmid and its variants, which are widely distributed in *A*. *baumanni* and the most common cause of resistance to gentamicin and tobramycin within this species^26^. In particular, pIH13 was 99% and 97% identical to pALWED1.8 and pAJOLS1.1, respectively^46^, both isolated from arctic permafrost. These plasmids and pIH13 harbored the *aadA27* gene that confers streptomycin and spectinomycin resistance. None of these *aadA27* genes represented gene cassettes, as was found in pRAY and related plasmids (Fig. 2). The Mob module of pIH13 also appeared to be conserved and no Rep module could be identified.

The plasmid sequence of pIH16 was highly similar to the previously described *A*. *baumannii* plasmids pD72-1 and pC2^30^, sharing almost identical backbones (Fig. S4). Replication and TA modules were conserved. Plasmid pIH16 encoded a truncated version of TonB-dependent outer membrane receptor gene and lacked the insertion sequence present in pD72-1 (IS*Aba*125). These plasmids also encoded a putative cholesterol-dependent cytolysin, named Septicolisyn.

Plasmids pIH17 and pIH19 are small plasmids, highly similar to pAB49-v1 from *A*. *nosocomialis* strain 178^47^. They all encoded a rolling circle replication protein and small hypothetical proteins (not shown). The biological importance of these small replicons remains to be determined and, for this reason, they are considered cryptic plasmids.

Plasmid pIH14 was homologous to a phage-related sequence that is present in the chromosome of *A*. *baumanii* AB0057^48^. Only the replication protein CDS as well as a small segment downstream of this CDS were homologous to plasmid pM131-10 of *Acinetobacter* sp. M13 (JX101639) (not shown).

Nucleotide sequence analysis of replicons phIH4, phIH5 and phIH10 showed that they all shared a high degree of sequence identity with putative phage sequences inserted in *Acinetobacter* chromosomes (Fig. S5). In particular, phIH10 was identical to a 6.8 kb region of *A*. *baumannii* AB0057^48^ and *A*. *baumannii* D36^49^. This region was also integrated into the chromosomes of several *A. baumannii* strains, such as AB5075-UW (CP008706 – unpublished), AYE^45^ and LAC-4^28^ in a repetitive manner. Given their similarities with phages together with the fact that no Rep module could be identified in their backbones, these three replicons were considered as prophages that probably excised during the plasmid isolation process.

### Analysis of deduced proteins involved in plasmid replication

In order to replicate as an extra-chromosomal element in a bacterial host, plasmids must have a functional, compatible and independent replication system. *In silico* analysis of the nosocomial collection sequences showed that 14 of the replicons harbored at least one Rep module (Table S3). It was not possible to identify any *rep* genes in the remaining five replicons, suggesting that they may have other replication mechanisms. Concerning the environmental plasmid collection, 25 Rep modules were identified within 25 plasmid contigs. Analysis of the predicted Rep domains of both plasmid collections revealed the predominance of the Rep_3 family (PF01051) over other Pfam families. Only 10 Rep module sequences (nine from the nosocomial and one from the environmental collection) could not be assigned to any Pfam domain (Table S3). The over-representation of the Rep_3 family was consistent with that found in all Rep proteins from *Acinetobacter* spp. plasmids in databases, since the Rep_3 domain proteins were the most abundant group (*ca*. 70%) (Fig. S6). *In silico* identification of putative replication origins was achieved in nosocomial collection plasmids harboring the Rep_3 domain (PF01051). Additional features surrounding the Rep_3 CDS could be identified, such as AT-rich regions, iterons and inverted repeats, all of them in different architectures (Fig. S7).

**Figure 3:**
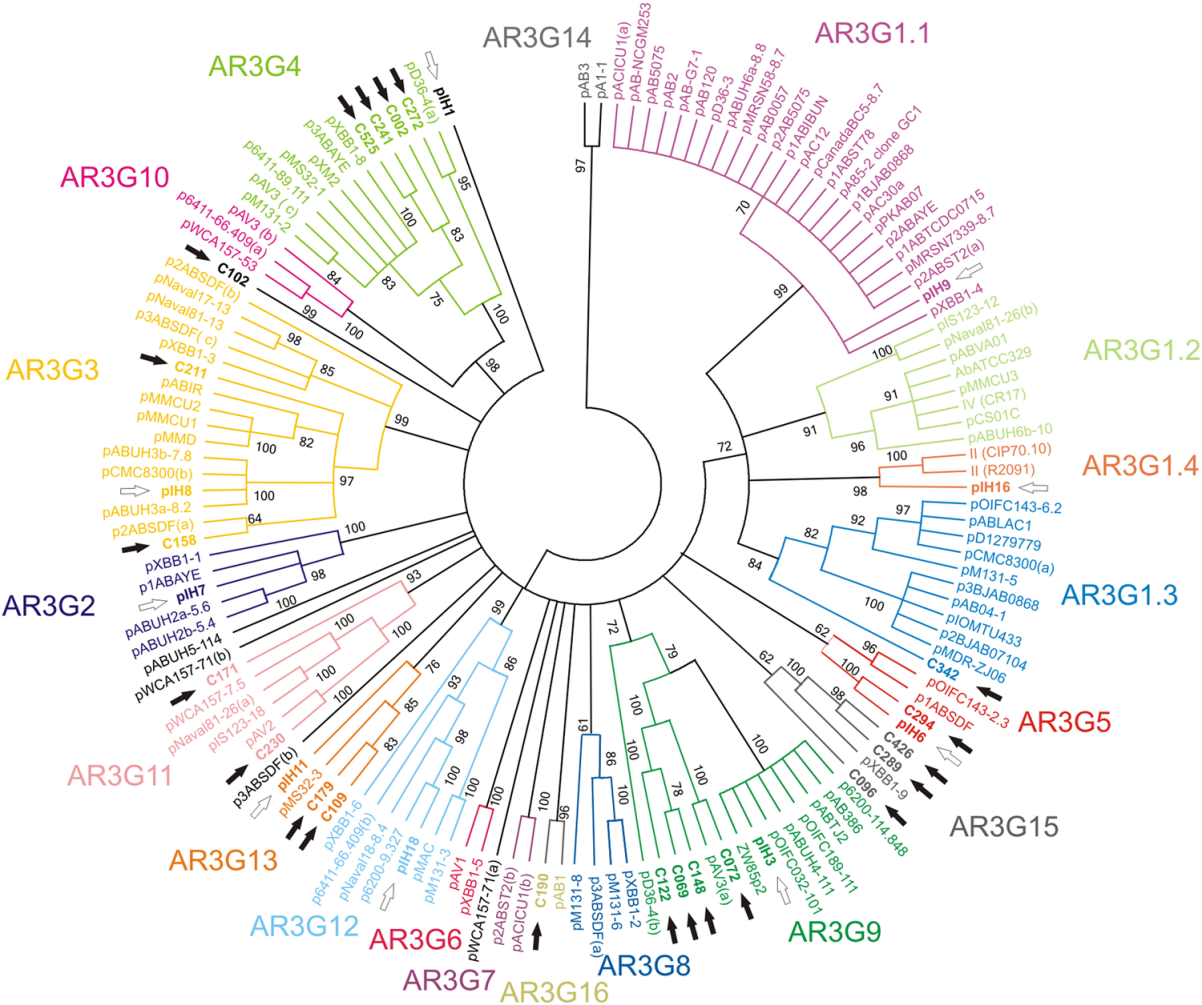
Phylogenetic tree of Rep_3 domain replication initiation proteins from *Acinetobacter* spp. plasmids. Resulting ML condensed (cut off value = 60) phylogenetic tree of Rep_3 domain (PF01051) proteins of all *Acinetobacter* spp. plasmids from databases and from the collections analyzed in this study. Topology was supported using bootstrap values of 100. Each of the 16 groups are marked in colors. Black arrows point the position of Rep_3 proteins of the nosocomial collection. White arrows show the position of Rep_3 proteins of the environmental plasmid contigs analyzed.

The phylogenetic analysis of Rep proteins belonging to the Rep_3 (PF01051) family was used to determine the evolutionary relationship of these proteins. All Rep_3 family proteins from *Acinetobacter* spp. plasmids from databases (Table S4) and both collections used in this study were aligned. A maximun likelihood (ML) tree was constructed, using Bootstrap values of 100 for topology support. Within the resulting tree, 16 statistically supported ***A****cinetobacter* **R**ep **3 G**roups (AR3G) could be established. Eight plasmids from the hospital collection according to their Rep_3 replication protein were classified within eight different groups: pIH9 into AR3G1.1, pIH16 into AR3G1.4, pIH7 into AR3G2, pIH8 into AR3G3, pIH6 into AR3G5, pIH3 into AR3G9, pIH18 into AR3G12 and pIH11 into AR3G13 (Fig. 3). Environmental plasmid contigs containing Rep_3 domain proteins were also classified into different groups, together with Rep of plasmids isolated from hospitals as well as from other environments. Comparative analysis of this phylogenetic study with those carried out by Bertini, *et al*.^50^ and Shintani, *et al*.^51^ revealed that seven groups defined in this study (AR3G 2, 4, 5, 7, 8, 12 and 16) were coincident with GR 11, 13, 1, 10, 7, 8 and 17, respectively. The AR3G1 group was subdivided into four different subgroups: AR3G1.1 and AR3G1.2, which included members of GR2; AR3G 1.3, that was coincident with GR3, and AR3G 1.4, which contained Rep proteins highly similar to GR4 and GR5. The AR3G3 group contained members of GR9, GR12, GR15 and GR18. The other groups defined in this study, AR3G6, AR3G9, AR3G10, AR3G11, AR3G13, AR3G14 and AR3G15 contained as yet unclassified Rep proteins. The remaining GR group included an *Acinetobacter* Rep_3 family protein, GR19, which was not considered in this study since its single member corresponded to a truncated sequence and therefore was not used for phylogenetic analysis.

Consensus sequences from defined groups were used in order to search for orthologous proteins in other genera beyond *Acinetobacter*. Analysis revealed that these Rep_3 proteins had orthologs in several genera, such as *Psychrobacter*, *Pseudomonas*, *Neisseria*, *Bacillus* and *Escherichia*.

In order to determine the functionality of putative predicted Rep_3 family Rep modules in an *A*. *baumannii* background, plasmids pIH7, pIH8 and pIH16 from the hospital collection were selected for further characterization. For this purpose, the predicted replication origins (Fig. S7) were cloned into an *Acinetobacter* non-replicative vector and further mobilized by conjugation into the *A*. *baumannii* A118 plasmid-free strain^52^. Transconjugant plasmid profiles evidenced that the constructed derivatives remained as independent replicons in the recipient cells, indicating that the proposed origins of replication identified *in silico* were correctly assigned (not shown).

### Stability modules

Different plasmid mechanisms involved in plasmid stability have been described in the literature, such as multimer resolution^53^, active partitioning^54^ and post-segregational killing, also known as TA systems or plasmid addiction systems^55,56^. Analysis of plasmid sequences revealed the presence of 15 and 70 CDS in the nosocomial and environmental collections, respectively, homologous to plasmid stability genes (Table S2). From the total, 28.4% of the sequences were homologous to genes belonging to active partitioning systems and 67.5% belonged to Type II TA systems such as BrnT/A, RelE/B, VapB/C, HicA/B, Yoeb/YefM and MazF/E. The remaining 3.5% corresponded to genes belonging to the StbABC system, which plays an important role not only in partition, but also in plasmid conjugation^57^.

### Conjugal transfer modules

**Figure 4:**
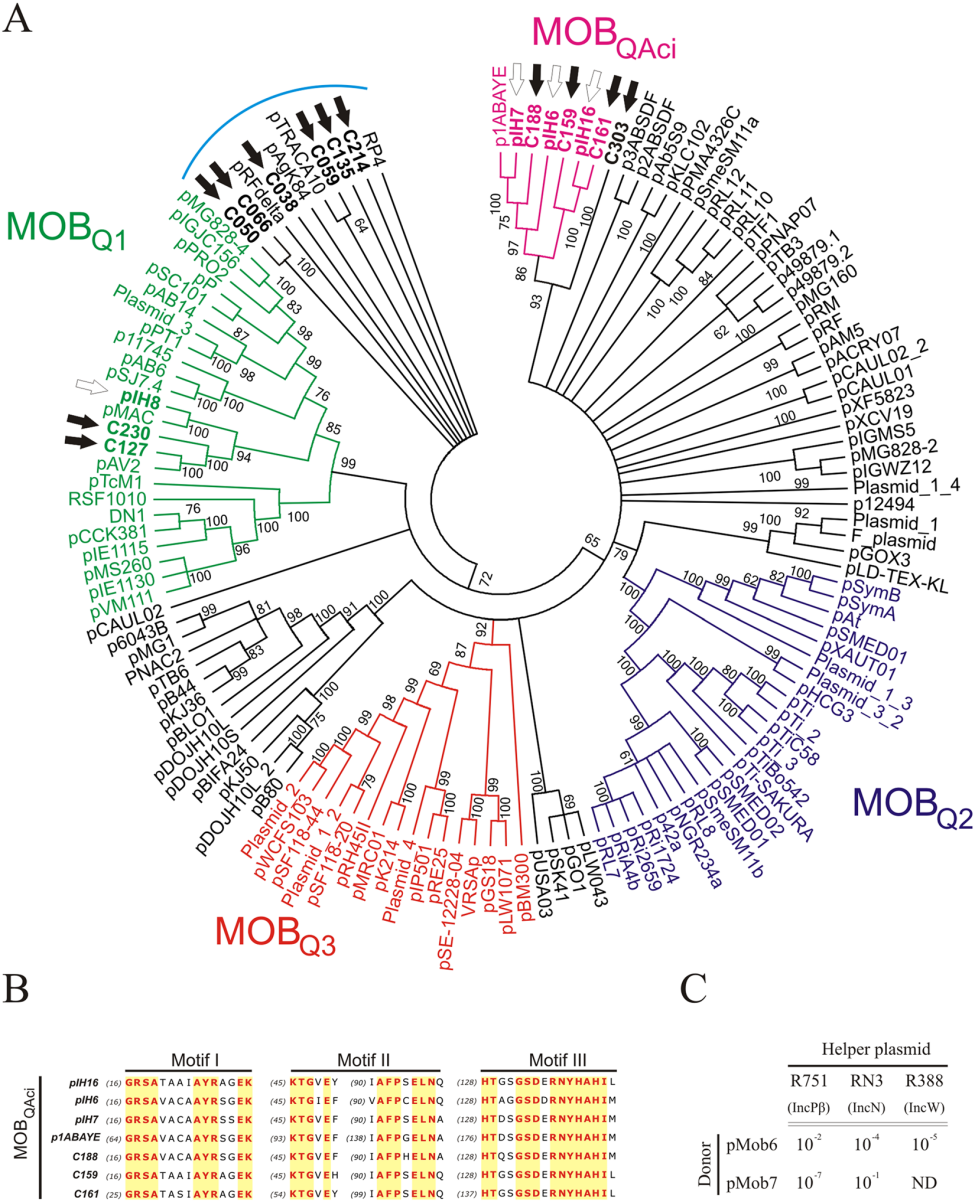
Analysis of plasmid relaxases belonging to the MOB_Q_ family. ML phylogenetic tree resulting from the 300 N-ter aminoacids of MOB_Q_ relaxases (Garcillán-Barcia *et al*.^42^) from database and this collection. (**A**) The MOB_Q_ tree shows the already described MOB_Q1_, MOB_Q2_ and MOB_Q3_ sub-families in different colors. White arrows indicate the position of MOB_Q_ relaxases from the nosocomial plasmid collection. Black arrows correspond to relaxases from environmental plasmid contigs. Relaxases from pIH6, pIH7. pIH16, C159, C161, C188 and p1ABAYE grouped together into a new subgroup named MOB_QAci_. (**B**) Alignment of MOB_QAci_ 300 N-ter aminoacid sequences shows three highly conserved and unique motifs when compared with those found in other MOB_Q_ subfamilies. Invariant aminoacids are shown in red over yellow. Relaxases that grouped together with RP4 plasmid (used as root, light-blue line) did not belong to the MOB_Q_ superfamily. (**C**) Conjugation frequencies of Mob constructions in the presence of different helper plasmids are expressed as the number of transconjugants per donor cell. ND, not detected.

Plasmid conjugation can contribute to the dissemination of genes mediating host adaptation to specific environmental niches. Therefore, characterization of conjugative/mobilizable plasmids contributes to the knowledge of gene dissemination and evolution within prokaryotic communities. The Mpf (Mating pair formation) genes (*trb* or *vir*) encode Type 4 Secretion System (T4SS) proteins that participate in establishing contact among cells, while Dtr (DNA transfer and replication) genes (*tra or mob*) are involved in DNA processing and replication. Transfer itself requires coupling of Dtr and Mpf systems. Plasmids that possess the complete sequence information for their own transfer are classified as self-transmissible. In contrast, the so-called mobilizable plasmids, despite possessing their own origin of transfer, still need helper functions (namely, T4SS) which are usually provided by a self-transmissible plasmid. *In silico* analysis of the hospital plasmid collection revealed that seven plasmids carried conjugal transfer modules. Despite the fact that one was predicted to be a conjugative plasmid (pIH2), its classification should be considered carefully, since the conjugal transfer phenotype depends not only on the genes encoded on the plasmid, but also on the genomic background of the host^58^. Five plasmids (pIH6, pIH7, pIH8, pIH13 and pIH16) were classified as putative mobilizable replicons. One of them, namely pIH1, could not be classified because the CDS encoded a truncated relaxase. Since closed circular replicons could not be obtained after sequencing the environmental plasmid collection, the analysis relied on *in silico* search of single genes involved in conjugation instead of whole conjugative modules. Results revealed that 25 contigs carried at least one conjugal transfer protein; of these, 15 encoded proteins associated with T4SS modules, suggesting that these contig sequences belonged to self-transmissible plasmids. In addition, 13 relaxase genes from 13 contigs could be identified (Table S5). Relaxases (Tra or Mob protein) play a key role in plasmid conjugation and were therefore proposed for transmissible plasmid classification^41,42^. Comparative phylogenetic analysis of the N-terminal amino acid sequences of the relaxases found in this study was carried out as proposed by Garcillán Barcia *et al*.^42^. Our results permitted to classify plasmid relaxases from the hospital (n = 6) and environmental (n = 10) collections into different MOB families and subfamilies: relaxases from pIH2 and contig C059 into MOB_F1_ (Fig. S8), from pIH13 into MOB_HEN_ (Fig. S9), from contigs C050 and C066 into MOB_P4_, and from contig C214 into MOB_P5_ (Fig. S10). Finally, relaxases from pIH6, pIH7, pIH8, pIH16 and contigs C127, C159, C161, C188, C230 and C303 could be classified as members of the MOB_Q_ super-family. In particular, relaxases from pIH8 and contigs C127 and C230 were classified into the MOB_Q1_ sub-family (Fig. 4A). Interestingly, relaxases from plasmids pIH6, pIH7, pIH16 and contigs C159, C161 and C188 grouped together into a MOB_Q_ subclade only comprising *Acinetobacter* spp. plasmids. Further analysis of these sequences revealed new conserved sequence motifs, suggesting they could be members of a new MOB_Q_ subfamily that was named MOB_QAci_ (Fig. 4B). Analysis of DNA sequences upstream of the MOB_QAci_ relaxases showed the putative origins of transfer (Fig. S11). Relaxases of contigs C038 and C135 could not be classified into the established groups. The relaxase encoded on contig C112 was not included in this analysis since its functional domains appeared to be truncated.

In order to determine whether plasmids carrying these novel MOB_QAci_ modules were capable of being mobilized by conjugative plasmids belonging to different Inc groups, a functional analysis of the modules present in pIH6 and pIH7 was carried out. For this purpose, Mob modules from both plasmids were cloned into a non-mobilizable vector and then transformed into bacteria already harboring different helper plasmids. Then, the ability to mobilize the constructed plasmids to the *E. coli* HB101 plasmid-free recipient strain was tested. Transfer assays showed that differences in conjugation frequencies were obtained when each Mob region was mobilized by different helper plasmids representing different Inc groups. Interestingly, despite pIH7 and pIH6 were closely related and belonged to the same MOB_QAci_ sub-family, their Mob regions were differentially mobilized by the same helper plasmid. Most remarkably, pIH7 Mob region was not able to mediate transfer by IncW plasmid R388, whereas the construct carrying the Mob region of pIH6 could be mobilized by R388 (Fig. 4C).

## Discussion

The ability of bacteria to colonize and adapt to different ecological niches can be explained by HGT MGE-mediated mechanisms, especially plasmids. Plasmids are responsible for the propagation of different traits, such as antibiotic resistance, specific degradation pathways, symbiosis and virulence, by promoting the transfer/exchange of genes within microbial populations. Therefore, studies focusing on plasmid maintenance and dissemination, acquisition and loss, will help reveal aspects of gene flux in ecosystems.

Species belonging to the genus *Acinetobacter* have a large pan-genome due to the presence of a great amount of dispensable genes^22^. This accessory genome is dynamic and consists of genes located in the chromosome as well as in mobile genetic elements, such as plasmids. Horizontal acquisition of information facilitates colonization of a great variety of environments and can explain the emergence of multi-resistant *Acinetobacter* spp. clones. Thus, the study of *Acinetobacter* spp. plasmid biology might be helpful to understand how these elements have become efficient vectors of genetic exchange.

In this study, two *Acinetobacter* spp. plasmid collections from different environments were sequenced and characterized, with special emphasis on comparative genomics and the description of modules specifying plasmid functions such as replication, stable maintenance and mobilization by conjugation. As a first step, the plasmid content of two different isolate collections, one representing nosocomial strains and the other environmental strains, was evaluated. Our results showed that most isolates from both collections carried plasmids. The sequenced replicons obtained had different sizes, and there was no obvious difference in plasmid size distribution of both collections.

Sequencing of the hospital plasmid collection showed little chromosomal contamination, which could be removed *in silico* using *A. baumannii* genomes as a reference. This was possible because of the relative homogeneity of the sample, which mostly consisted of isolates of the species *A. baumannii*. The remaining plasmid scaffolds could be completed *in silico* using computer tools due to the low amount of repetitive and insertion sequences in the sample. Sequencing of the environmental plasmid collection resulted in longer sequence reads and a larger amount of sequences. The average size of contigs exceeded that of contigs from the hospital collection, suggesting that larger plasmids were obtained from environmental habitats. *In silico* extraction of chromosomal information using reference genomes did not give completely satisfactory results. This was due to the heterogeneity of the sample, which was composed of several species of the genus *Acinetobacter*, and to the fact that appropriate genomes of other *Acinetobacter* species are not available in databases. Thus, to continue the analysis, only contigs exclusively carrying plasmid genes were selected. Although this strategy allowed recovery of an acceptable amount of plasmid contigs, accessory plasmid sequence information might have got lost.

Comparative genomic analysis results showed that closed replicons had conserved backbones. This was the case of plasmids pIH2 and pIH13. Plasmid pIH2 possesses a conserved IncN architecture that is highly similar to the already described IncN2 plasmids pJIE137 and p271A. All these plasmids encode a novel RepA protein and are therefore considered as a new class of IncN plasmids, named IncN2^40^. Interestingly, unlike typical IncN-like plasmids, pIH2 did not carry any resistance genes. This finding substantiates that the IncN-like backbone is highly conserved and an efficient vehicle for the transmission of information when antibiotic resistance genes get inserted into it.

Plasmid pIH13 was also similar to other plasmids from databases, showing 99% and 97% identity to plasmids pALWED1.8 and pAJOLS1.1, respectively^46^. The typical architecture of these replicons was also found in pRAY and its variants, which have so far only been identified in *Acinetobacter* species and are responsible for an aminoglycoside-resistant phenotype. Kurakov *et al*.^46^ also identified plasmids almost identical to pALWED1.8 in ancient and modern strains of *Acinetobacter* spp. as well as in the *A. parvus* strain CM11 isolated from mice intestine. These findings revealed the ubiquity of these pRAY-related plasmids and the conservation of a highly specific plasmid structure among different *Acinetobacter* species.

Due to the central role of plasmids in the evolution of bacteria, the comparative analysis of Rep proteins is of considerable importance not only to understand how bacteria acquired information, but also to potentially predict the host range of plasmids. Replication initiation protein analysis showed that the Rep_3 family domain proteins were most frequently found in the collections studied here and *Acinetobacter* spp. plasmids from databases. This finding could indicate that plasmids carrying the Rep_3 module may represent vectors for the transfer of genetic information among *Acinetobacter* spp. from different environments. The fact that Rep_3 domain proteins were found in different species, families and even phyla, suggests that *Acinetobacter* plasmids carrying Rep_3 proteins may be able to replicate in non-related bacteria. This can partly explain the large pan-genome present in *Acinetobacter* spp., since plasmids from diverse origins carrying various genes can reach members of this genus by HGT and stably persist and replicate, enlarging the set of accessory genes.

Plasmid conjugation is probably the most important HGT mechanism, and therefore plays a key role in the spread of genetic information. *In silico* analysis of conjugation proteins found in both collections led to the identification of 19 relaxase proteins, from which 16 could be classified into different families and subfamilies according to Francia *et al*.^41^ and Garcillán-Barcia *et al*.^42^. Interestingly, a new subgroup exclusively composed of relaxases from *Acineotbacter* spp. plasmids with unique characteristics within their relaxase N-terminal domain motifs was identified in the present study. However, functional analysis of Dtr modules from two members of this new MOB_QAci_ sub-family showed differences in conjugation frequencies when mobilized by the same helper plasmid. These results, together with the information obtained from sequence alignments of the complete relaxase proteins, suggest that these MOB_QAci_ relaxase N-terminal domains would not be responsible for the recognition of the mobilizable plasmid by the coupling proteins (CP) provided in *trans* by helper plasmids. For this reason, plasmids belonging to the same MOB family may highlight distinct transfer properties even when mobilized from the same host bacterium. Accordingly, prediction of transfer properties of MOB_QAci_ plasmids based only on the corresponding N-terminal relaxase domains would not be reliable.

Most studies on plasmid diversity have focused on accessory genes encoding selected host-beneficial traits, such as resistance determinants or xenobiotic degradation genes of biotechnological importance. Fewer studies have explored plasmid diversity and gene content using plasmid isolation methods that are not based on a plasmid-encoded accessory phenotype^59^. Despite many studies describe plasmids involved in the spread of resistance genes among clinical *Acinetobacte*r strains^23–33^, few have characterized the biology of plasmids in *Acinetobacter* species. For this reason, the present study focused exclusively on plasmid-specific functions of *Acinetobacter* spp. from both environmental and nosocomial bacteria. The fact that the conjugative transfer modules and the replication initiation proteins found in this work are shared with plasmids from other bacterial genera reinforces the idea that plasmids can act as key “vehicles”, spreading different traits and contributing to bacterial evolution.

The information gathered in the present study not only increases the current knowledge on *Acinetobacter* plasmid biology, but also provides evidence that *Acinetobacter* plasmids might have evolved to become specialized vectors for the transfer of genetic information among species of the same genus, as well as among unrelated bacteria. Analysis of plasmid functional modules contributes to the understanding of the dynamics of *Acinetobacter* genomic evolution towards its establishment as an important nosocomial pathogen.

## Methods

### Bacterial Collections

The bacterial strains and plasmids used in this study are listed in Table S6. Two different collections were assessed. The nosocomial collection consisted of 64 *Acinetobacter* spp. bacteria isolated from five different Buenos Aires hospital environments (patients, surfaces, equipment and furniture). All nosocomial bacteria were isolated and identified under the National Nosocomial Infection Surveillance System (NNIS) and the National Epidemiological Surveillance System for Hospital Infections (SIVENIH) (http://www.vihda.gov.ar/sitio%20vihdaii/vihda/vigilancia.asp) protocol criteria.

The environmental collection consisted of 59 *Acinetobacter* spp. bacteria isolated from soil and water samples from La Plata city ground. Samples were enriched in Baumann´s enrichment medium^60^ and then plated in Leeds Acinetobacter Medium (LAM)^61^ without antibiotic supplementation. Selected colonies were subjected to Gram staining, motility, oxidase test and genus identification by MALDI-TOF spectrophotometry^62^. Gram negative, non-motile and oxidase-negative isolates were used to perform *in situ* lysis gel electrophoresis in order to identify isolates harboring plasmids. Finally, 59 *Acinetobacter* spp. isolates were selected.

*Escherichia coli* as well as *Acinetobacter* spp. strains were grown on Luria–Bertani (LB) at 37 and 30 °C, respectively.

### Plasmid profiles

Plasmid profile analyses of bacteria belonging to the environmental collection were carried out as previously described by Pistorio *et al*.^38^. Plasmid profiles of bacteria belonging to the nosocomial collection were obtained by *in situ* lysis gel electrophoresis as described by Eckhardt^37^ with some modifications: overnight colonies were resuspended in 15 µl of lysozyme mixture and then loaded into 0.8 agarose, 1% SDS gel slots. The samples were electrophoresed for 60 min at 40 V until complete cell lysis was evidenced, and then for 1.5 h at 80 V. Agarose gels were washed with distilled water several times and then stained with ethidium bromide.

### DNA isolation and purification

Plasmids from the nosocomial collection were isolated as described by Kieser^63^, whereas plasmids from the environmental collection were isolated as described by Jouanin *et al*.^64^. After isolation, all plasmids from the nosocomial collection were pooled together conforming one nosocomial sample. Plasmids isolated from the environmental collection were also pooled together conforming one environmental sample. Both samples were separately subjected to isopycnic ultracentrifugation in a CsCl gradient, as described by Sambrook *et al*.^65^.

### DNA sequencing and bioinformatics tools

DNA samples obtained after ultracentrifugation were separately sequenced using MiSeq Illumina technology at the Center for Biotechnology (CeBiTec) in Bielefeld, Germany. The reads obtained were assembled using GS de novo Assembler software (gs Assembler, version 2.8, Roche). Plasmid finishing was accomplished using the CONSED^66^ software package. Complete closed replicons were automatically annotated by the genome annotation system GenDB^39^ and manually curated. Genes were annotated using the best BLASTP hit at the National Center for Biotechnology Information (NCBI) database (http://www.ncbi.nlm.nih.gov/). Replicon sequences were submitted to GenBank and registered under Bioproject ID PRJEB22129.

Sequence alignment and graphic comparison were performed using the genome comparison visualizer Easyfig^67^.

### Functional analysis

In order to confirm bioinformatics function assignment, experimental functional analysis was carried out for putative origins of replication, including replication initiation proteins (Rep), and mobilization (Mob) modules. Mob and Rep modules were cloned into pK18 and pK18*mob*, respectively. Inserts were obtained by PCR using kappa Hi Fi (Kappa Biosystems) polymerase and ligated to *Sma*I (Promega) digested vectors. Ligation mixture was used to transform competent DH5α cells and transformants were selected in agar plates containing LB supplemented with 50 µg/ml Kanamycin and X-gal. Transformants containing inserts were checked by PCR using universal primer sets. Clones containing Mob modules were isolated and further transformed into DH5α cells already containing helper plasmids R388^68^, RN3^69^ or R751^70^. All primers used in this study are listed in Table S7.

### Mating assays

Bacterial matings were performed as described by Simon *et al*.^71^. In brief, LB liquid cultures were grown at 37 °C under agitation until early exponential stage of donor cells and late exponential stage of recipient cells. Both cultures were mixed (1:1) into 1.5 tubes and centrifuged at 640 g for 8 min. Pellets were gently resuspended into 50 µl LB, carefully plated on LB agar plates and grown overnight at 37 °C. Cells were resuspended in 1 ml LB and plated on LB plates supplemented with the corresponding antibiotics for transconjugant selection. Frequencies were calculated as number of transconjugants per number of recipient cells. Transconjugants were evaluated by *in situ* gel electrophoresis type gels. Co-integrates were never visualized in any of the experiments performed.

### Phylogenetic analysis

Protein alignments were carried out using ClustalW^72^ default parameters. Plasmids were classified according to their relaxase proteins as described by Garcillán-Barcia *et al*.^42^. MOB families and subfamilies were also defined as in Garcillán-Barcia *et al*.^42^. MOB relaxase and Rep_3 family phylogenetic analysis was carried out by maximun likelihood (ML) using PhyML 20131016^73^. The best evolutionary model for each multiple alignment was established by ProtTets 2.4^74^. Bootstrap values of 100 were chosen for ML analysis. Rep_3 database sequences used for phylogenetic tree calculation are listed in Table S4.

## Electronic supplementary material

Supporting information
