# Supplementary material for: Comparative genomic analysis of Acinetobacter spp. plasmids originating from clinical settings and environmental habitats
Source: Sci Rep. 2018 May 17;8:7783. doi: 10.1038/s41598-018-26180-3 (PMC5958079; doi:10.1038/s41598-018-26180-3)
Supplement: Supplementary file 1 — Supporting information [file 41598_2018_26180_MOESM1_ESM.pdf]

Supporting information

Comparative genomic analysis of *Acinetobacter* spp. plasmids originating from clinical settings and environmental habitats

Ileana P. Salto[1], Gonzalo Torres Tejerizo[1,2], Daniel Wibberg[2], Alfred Pühler[2], Andreas Schlüter[2], and Mariano Pistorio[1*]

[1] IBBM (Instituto de Biotecnología y Biología Molecular), CCT-CONICET-La Plata, Departamento de Ciencias Biológicas, Facultad de Ciencias Exactas, Universidad Nacional de La Plata, Calles 47 y 115 (1900) La Plata, Argentina.

[2] Center for Biotechnology (CeBiTec), Bielefeld University, Genome Research of Industrial Microorganisms, Universitätsstr. 27, D-33615 Bielefeld, Germany.

* Corresponding author: Mariano Pistorio

Phone: +54-221-422-9777 ext. 142

Fax: +54-221-422-3409 ext. 56

E-mail: pistorio@biol.unlp.edu.ar

Table S1. General environmental plasmid sequence features

Contig Name	Size(pb)	CDS	CDS with predicted functions							
			Rep	Man	Con	MGE	Res	BMRR	HP	Other
C002	30093	27	1	2		2			6	16
C003	20868	22		2					3	17
C006	889	2				2				
C030	11721	12			8				4	
C033	1136	1		1						
C034	1004	2		1					1	
C038	8644	5			2				2	1
C039	16756	21		2			2	2	8	7
C046	13445	16			2				11	3
C047	2466	1			1					
C048	3206	3			3					
C049	4065	4			2				2	
C050	13843	17		2	1		1		12	1
C052	19265	23		2		1	5		9	6
C057	20866	21			8		4		8	1
C058	6359	5		1					4	
C059	13881	11			2			3	5	1
C060	10049	14		3				1	10	
C063	3891	4			2				2	
C064	3201	3			3					
C065	2469	1			1					
C066	30076	29		2	3		2	3	17	2
C069	4361	6	1	1		1			3	
C070	6566	10		2		1			4	3
C072	4321	6	1	1		2			2	
C075	2414	3		2						1
C095	6847	9	1			1		1	6	
C096	8773	8	1	1		1			5	
C097	1387	3		2						1
C099	4849	8		2				1	5	
C100	1725	3		1					1	1
C101	6068	9		2				1	4	2
C102	9053	7	1						5	1
C108	8600	13		2				1	4	6
C109	10491	11	1						9	1
C112	5427	8			2				4	2
C122	19378	23	1	3		5		3	6	5
C127	12284	13			1			3	8	1
C135	14368	10		1	1	1			6	1
C136	929	1		1						
C148	5757	7	1	3					2	1

Table S1. General environmental plasmid sequence features (Continue)

Contig Name	Size(pb)	CDS	CDS with predicted functions							
			Rep	Man	Con	MGE	Res	BMRR	HP	Other
C158	10731	12	1					2	8	1
C159	6609	11		2	1			1	6	1
C161	5860	7			1				4	2
C171	6403	6	1					1	4	
C178	2038	5		2					3	
C179	8018	11	1						10	
C180	1992	4		1				1	2	
C188	6347	5			1				3	1
C190	9238	11	1						10	
C211	4718	6	1					1	3	1
C212	4881	8		2					5	1
C214	9126	10		3	1	2			3	1
C226	6412	9		2		3	2		1	1
C230	10183	10	1		1				7	1
C231	7597	11			2				9	
C239	2092	1		1						
C241	779	1	1							
C250	5856	7	1					1	5	
C252	2190	3		2					1	
C272	5162	5	1	2					1	1
C281	3810	7		2					2	3
C289	8300	12	1					1	9	1
C290	2213	2	1						1	
C294	5433	6	1		1		1		3	
C300	2395	6		2					3	1
C303	4550	6		1	1				4	
C304	4076	7		1		2	2		1	1
C309	4002	9		2					6	1
C334	5523	7	1					1	5	
C338	2093	1		1						
C342	2583	2	1						1	
C360	1298	2			1				1	
C426	1491	1	1							
C465	1136	1		1						
C525	780	1	1							
C681	335	1		1						

Rep, plasmid replication; Mant, plasmid maintenance; Con, plasmid conjugation; MGE, mobile genetic element; Res, Resistance; BMRR, DNA binding /modification /restriction /recombination; HP, hypothetical proteins; Other, other predicted functions.

Table S2. Predicted plasmid maintenance proteins present in nosocomial and environmental plasmid sequences.

Replicon/ contig	Protein name	Pfam Name	Pfam Code	GeneBank Accesion	Genus	% Identity
pIH1	Plasmid stabilization protein ParE	ParE_toxin	PF05016	KJP73628.1.1	*E.hormaechei*	100
	Virulence associated protein VagC	MazE_antitoxin	PF04014	EUM08956.1	*Enterobacter* sp.	100
	PilT domain protein	PIN	PF01850	WP_032662169.1	*E.cloacae*	99
	StbA	StbA	PF06406	WP_045328477.1	*E.cloacae*	100
	Plasmid stability mediator	Plasmid_ stabB	PF10784	WP_045325846.1	*E.cloacae*	99
pIH2	StbA	-	-	AEY80235.1	*K.pneumoniae*	99
	Plasmid stabilization protein StbB	-	-	YP_004765036.1	*Enterobacteriaceae*	100
	StbC	-	-	YP_004765035.1	*E. coli*	100
pIH3	Partition protein ParA	ParA	PF10609	WP_000770709.1	*A. calcoaceticus / baumannii*	100
pIH8	Addiction module, toxin	YafQ_toxin	PF15738	WP_000176412.1	*Acinetobacter* sp.	100
	Addiction module, antitoxin	RelB	PF04221	WP_0051430357.1	*Acinetobacter* sp.	100
pIH11	Hypothetical protein	BrnT_toxin	PF04365	EEY94627.1	*A. johnsonii*	93
	Antitoxin	BrnA_antitoxin	PF14384	WP_061855757.1	*Acinetobacter* sp.	94
pIH16	Membrane protein	Brn T_toxin	PF04365	EKK14970.1	*A. baumannii*	100
	Antitoxin	BrnA_antitoxin	PF14384	WP_000438826.1	*A. baumannii*	100
C002	Partition protein	ParBc	PF02195	WP_067869890.1	*Acinetobacter* sp.	97
	Partition protein	CbiA<br>MipZ	PF01656<br>PF09140	WP_074947363.1	*A. bohemicus*	98
C003	Addiction module, antitoxin	HTH_3	PF01381	OIG81307.1	*A. baumannii*	99
	Addiction module, toxin	-	-	KQD29442.1	*A. pittii*	96
C033	Partition protein ParA	CbiA	PF01656	AOB42321.1	*A. baumannii*	99
C034	Partition protein ParB	-	-	WP_004282012.1	*A. lwoffii*	98
C039	Toxin YoeB	YoeB_toxin	PF06769	WP_004683136.1	*A. parvus*	91
	Antitoxin	PhdYeFM_antitox	PF02604	WP_010111585.1	*Acinetobacter* sp.	90
C050	Partition protein	CbiA	PF01656	WP_071212513.1	*A. baumannii*	96
	Partition protein ParB	KorB<br>ParBc	PF08535<br>PF02195	WP_068550223.1	*A.pittii*	84
C052	Addiction module, antitoxin	ParE_toxin	PF05016	WP_005021147.1	*Acinetobacter* spp.	95
	Plasmid stabilization protein	PhdYeFM_antitox	PF02604	WP_053578851.1	*Acinetobacter* sp.	96
C058	Partition protein	CbiA	PF01656	WP_071212513.1	*A. baumannii*	69
C060	ATPase	CbiA<br>MipZ	PF01656<br>PF09140	WP_000807825.1	*A. baumannii*	45
	Toxin YoeB	YoeB_toxin	PF06769	SFT25128.1	*A. bohemicus*	97
	YefM (antitoxin of YoeB)	PhdYeFM_antitox	PF02604	EPH32698.1	*A. guillouiae*	92
C066	Partition protein ParB	KorB_C<br>ParBc	PF06613<br>PF02195	WP_068550223.1	*A. pittii*	97
	Partition protein	CbiA<br>MipZ	PF01656<br>PF09140	WP_071212513.1	*A.baumannii*	98
C069	Partition protein ParA	CbiA<br>MipZ	PF01656<br>PF09140	WP_008306811.1	*Acinetobacter* spp.	98
C070	YefM (antitoxin of YoeB)	PhdYeFM_antitox	PF02604	EPH32698.1	*A.guillouiae*	91
	Toxin Txe/YoeB	YoeB_toxin	PF06769	WP_005264103.1	*Acinetobacter* sp.	89
C072	ParA	CbiA<br>MipZ	PF01656<br>PF09140	EEY91718.1	*A. junii*	100
C075	PIN domain protein	PIN	PF01850	EET81748.1	*A. radioresistens*	97
	Virulence associated protein B	MazE_antitoxin	PF04014	WP_034700678.1	*Acinetobacter* sp.	96
C096	Partition protein ParA	-	-	KRJ26257.1	*A.baumannii*	98
C097	HicA	HicA_toxin	PF07927	EZQ01110.1	*Acinetobacter* sp.	99
	Antitoxin HicB	HicB	PF05534	WP_010117354.1	*Acinetobacter* sp.	97
C099	Addiction module, toxin	-	-	WP_044109848.1	*Acinetobacter* sp.	100
	Addiction module, antitoxin	HTH_3	PF01381	WP_060875721.1	*A.baumannii*	99
C100	Addiction module, antitoxin	HTH_3	PF01381	EFF84300.1	*A.haemolyticus*	100

Table S2. Predicted plasmid maintenance proteins present in nosocomial and environmental plasmid sequences. (Continue)

Replicon/ contig	Protein name	Pfam Name	Pfam Code	GeneBank Accesion	Genus	% Identity
C101	Toxina RelE	ParE_toxin	PF05016	EEY85385.1	*A.radioresistens*	100
	Regulador transcripcional	HTH_3<br>MqsA_antitoxin	PF01381<br>PF15731	WP_000369781.1	*Acinetobacter* spp.	100
C108	Toxin	HigB-like_toxin	PF05015	EIG27648.1	*H. paraphrohaemolyticus*	68
	Addiction module, antitoxin. HigA family	HTH_3	PF01381	WP_067724800.1	*Acinetobacter* sp.	93
C122	Partition protein ParA	CbiA - MipZ	PF01656<br>PF09140	WP_008306811.1	*Acinetobacter* spp.	99
	Addiction module, antitoxin. RelB	-	-	ODI90385.1	*A.pittii*	94
	Addiction module, antitoxin	HTH_3	PF01381	WP_068912099.1	*Acinetobacter* sp.	98
C135	CobQ/CobB/MinD/ParA	CbiA<br>MipZ	PF01656<br>PF09140	WP_004641746.1	*A.haemolyticus*	47
C136	Antitoxin	-	-	WP_067731965.1	*Acinetobacter* sp.	71
C148	ParA	CbiA<br>MipZ	PF01656<br>PF09140	EIM38468.1	*Acinetobacter* sp.	98
	VapB	MazE_antitoxin	PF04014	YP_009070348.1	*Acinetobacter sp.*	99
	VapC	PIN	PF01850	WP_012268403.1	*Acinetobacter* spp.	95
C159	Antitoxin igA-2	MqsA_antitoxin<br>HTH_3	PF15731<br>PF01381	KZA76063.1	*A. baumannii*	95
	Transcriptional regulator	-	-	KHW80295.1	*A. baumannii*	97
C178	Toxin YafQ	YafQ_toxin	PF15738	EPR82854.1	*A. gerneri*	97
	Antitoxin RelB/DinJ	RelB	PF04221	WP_005254175.1	*Acinetobacter* spp.	100
C180	Toxin RelE	ParE_toxin	PF05016	EEY85385.1	*A. radioresistens*	100
C212	Transcriptional represor RelB	RelB	PF04221	WP_074947748.1	*A. bohemicus*	99
	Plasmid stabilization protein	ParE_toxin	PF05016	WP_074947749.1	*A. bohemicus*	
C214	Hypothetical protein	RelE	PF06296	WP_004977497.1	*A. towneri*	100
	Transcriptional regulator	HTH_3	PF01381	WP_000369782.1	*Acinetobacter* spp.	100
	Partition protein ParA	CbiA<br>MipZ	PF01656<br>PF09140	EPR85282.1	*A. gerneri*	100
C226	HicA	HicA_toxin	PF07927	EPR85295.1	*A. gerneri*	100
	Antitoxin HicB	HicB	PF05534	WP_004812232.1	*Acinetobacter* spp.	100
C239	Partition protein	CbiA<br>MipZ	PF01656<br>PF09140	WP_074947363.1	*A.bohemicus*	99
C252	PIN domain protein	PIN	PF01850	EFF84337.1	*A.haemolyticus*	99
	Virulence associated protein B	MazE_antitoxin	PF04014	WP_046737959.1	*Acinetobacter* sp.	99
C272	Partition protein	ParBc	PF02195	WP_035270317.1	*Acinetobacter* sp.	98
	Partition protein	CbiA<br>MipZ	PF01656<br>PF09140	WP_035270320.1	*Acinetobacter* sp.	99
C281	Addiction module, antitoxin	HTH_3	PF01381	WP_068912099.1	*Acinetobacter* sp.	99
	Addiction module, toxin	-	-	WP_000286964.1	*Acinetobacter* spp.	99
C300	VapB	MazE_antitoxin	PF04014	WP_012268402.1	*A.venetianus*	91
	VapC toxin. PIN domain ribonuclease	PIN	PF01850	OJU89597.1	*Acinetobacter* sp.	93
C303	Antitoxin. Ribbon-helix-helix domain protein.	-	-	ELW84420.1	*Acinetobacter* sp.	72
C304	Antitoxin AbrB	MazE_antitoxin	PF04014	EFF84336.1	*A. haemolyticus*	91
C309	YefM (antitoxin of YoeB)	PhdYeFM_antitox	PF02604	EPH32698.1	*A. guillouiae*	94
	Toxin Txe/YoeB	YoeB_toxin	PF06769	WP_005264103.1	*Acinetobacter* sp.	90
C338	Partition protein	CbiA<br>MipZ	PF01656<br>PF09140	WP_067724373.1	*Acinetobacter* sp.	98
C465	Partition protein ParA	CbiA	PF01656	AOB42321.1	*A. baumannii*	99
C681	Addiction module, antitoxin	HTH_3	PF01381	EFF84300.1	*A. haemolyticus*	100

Table S3. Predicted Rep proteins present in nosocomial and environmental plasmids sequences.

Replicon / Contig	Protein name	Pfam Name	Pfam Code	GeneBank Accesion N	Genus	% Indentity
pIH1	Replication initiation protein	IncFII_repA	PF02387	WP_045328476.1	*E.cloacae*	100
	RepB	Rep_3	PF01051	WP_029403849.1	*Enterobacteriaceae*	100
	Plasmid F RepC-like	-	-	SAU96169.1	*K. pneumoniae*	85
pIH2	Rep_A	RepA_C	PF04796	WP_000807122.1	*Enterobacteriaceae*	100
pIH3	RepB	Rep_3	PF01051	WP_000818856.1	*Acinetobacter*	100
pIH6	RepB	Rep_3	PF01051	WP_065996348.1	*A.baumannii*	99
pIH7	RepB	-	-	WP_050042213.1	*A.parvus*	100
pIH8	RepB	Rep_3	PF01051	WP_005804946.1	*Acinetobacter* sp.	100
pIH9	Replication initiation protein	-	-	EYC96371.1	*A. baumannii*	99
	DNA replication protein	HTH_17	PF12728	WP_068553181.1	*A. pittii*	98
	RepB	Rep_3	PF01051	ADX94286.1	*A. baumannii*	99
phIH10	Replication protein	Rep_trans	PF02486	WP_000505023.1	*A. baumannii*	100
pIH11	Replicase	Rep_3	PF01051	WP_075041377.1	*A. radioresistens*	98
	DNA replication protein	-	-	EJW84558.1	*W. bancrofti*	95
pIH14	Rep63	-	-	KQK32674.1	*A. baumannii*	99
pIH16	RepB	Rep_3	PF01051	WP_033107831.1	*A. baumannii*	100
	DNA replication protein	-	-	KOR09926.1	*Acinetobacter* sp.	51
pIH17	Rep63	-	-	EMT93808.1	*A. baumannii*	86
pIH18	RepB	Rep_3	PF01051	WP_005133531.1	*A. baumannii*	100
	DNA replication protein	-	-	KQK32652.1	*A. baumannii*	100
pIH19	Rep63	-	-	WP_057078279.1	*A. baumannii*	100
C002	RepB family plasmid replication initiator protein	Rep_3	PF01051	WP_067869884.1	*Acinetobacter* sp.	96
C069	Initiator RepB protein	Rep_3	PF01051	WP_064095420.1	*Acinetobacter* sp.	95
C072	Initiator RepB protein	Rep_3	PF01051	EKA68794.1	*A. baumannii*	100
C095	Plasmid replicase, partial	Replicase<br>PriCT-1<br>HTH	PF09090<br>PF08708<br>PF13384	WP_010591570.1	*Acinetobacter* spp.	86
C096	RepB family plasmid replication initiator protein	Rep_3	PF01051	WP_057692638.1	*A. baumannii*	72
C102	RepB family plasmid replication initiator protein	Rep_3	PF01051	WP_004967300.1	*Acinetobacter* spp.	70
C109	RepB family plasmid replication initiator protein	Rep_3	PF01051	WP_039624411.1	*A. harbinensis*	91
C122	Initiator RepB protein	Rep_3	PF01051	WP_064095420.1	*Acinetobacter* sp.	96
C148	Initiator RepB protein	Rep_3	PF01051	WP_064095420.1	*Acinetobacter* sp.	93
C158	Protein involved in initiation of plasmid replication	Rep_3	PF01051	SDY87313.1	*A. kyonggiensis*	99
C171	RepB family plasmid replication initiator protein	Rep_3	PF01051	WP_004641847.1	*A. haemolyticus*	72
C179	RepB family plasmid replication initiator protein	Rep_3	PF01051	WP_004676211.1	*A. parvus*	91
C190	RepB family plasmid replication initiator protein	Rep_3	PF01051	WP_005166868.1	*Acinetobacter* sp.	83
C211	RepB family plasmid replication initiator protein	Rep_3	PF01051	WP_004734502.1	*Acinetobacter* spp.	97

Table S3. Predicted Rep proteins present in nosocomial and environmental plasmids sequences. (Continue)

Replicon / Contig	Protein name	Pfam Name	Pfam Code	GeneBank Accesion N	Genus	% Indentity
C230	RepB family plasmid replication initiator protein	Rep_3	PF01051	WP_005244554.1	*Acinetobacter* sp.	100
C241	RepB family plasmid replication initiator protein	Rep_3	PF01051	WP_067724374.1	*Acinetobacter* sp.	100
C250	Plasmid replicase	Replicase PriCT-1	PF03090 PF13384	WP_068538202.1	*A. pittii*	68
C272	RepB family plasmid replication initiator protein	Rep_3	PF01051	WP_035270863.1	*Acinetobacter* sp.	99
C289	RepB family plasmid replication initiator protein	Rep_3	PF01051	WP_005022088.1	*A. radioresistens*	98
C290	RepB family plasmid replication initiator protein	-	-	WP_004845629.1	*Acinetobacter* spp.	100
C294	RepB family plasmid replication initiator protein	Rep_3	PF01051	WP_065996348.1	*A. baumannii*	100
C334	Plasmid replicase	Replicase PriCT-1 HTH	PF09090 PF08708 PF13384	WP_034597285.1	*Acinetobacter sp.*	74
C342	RepB family plasmid replication initiator protein	Rep_3	PF01051	WP_004845629.1	*Acinetobacter* spp.	76
C426	RepB family plasmid replication initiator protein	Rep_3	PF01051	WP_005022088.1	*A. radioresistens*	99
C525	RepB family plasmid replication initiator protein	Rep_3	PF01051	WP_067724374.1	*Acinetobacter* sp.	100

Table S4. Rep_3 domain protein from database used in phylogenetic analysis.

Protein	GenBank Accesion N°	Plasmid	Genus/Species	Protein	GeneBank Accesion N°	Plasmid	Genus/Species
Rep_3	WP_000818857.1	p6200-114.848	*A. baumannii*	Rep_3	WP_000987942	pNaval17-13	*A. baumannii*
Rep_3	WP_038350249	p6200-9.327	*A. baumannii*	Rep_3	WP_002011482.1	pNaval18-8.4	*A. baumannii*
Rep_3	WP_001208776.1	pCS01C	*A. baumannii*	Rep_3	WP_000185726.1	pNaval81-26(a)	*A. baumannii*
Rep_3	WP_001208776.1	IV(CR17)	*A. baumannii*	Rep_3	WP_001208779	pNaval81-26(b)	*A. baumannii*
Rep_3	WP_000534216.1	pIOMTU433	*A. baumannii*	Rep_3	WP_000987942	pNaval81-13	*A. baumannii*
Rep_3	WP_001205343	pA1-1	*A. baumannii*	Rep_3	WP_000818857	pOIFC032-101	*A. baumannii*
Rep_3	WP_001205343.1	p2AB5075	*A. baumannii*	Rep_3	WP_000711927.1	pOIFC143-2.3	*A. baumannii*
Rep_3	WP_000818857.1	pAB386	*A. baumannii*	Rep_3	WP_000845851	pOIFC143-6.2	*A. baumannii*
Rep_3	WP_000534216.1	pAB04-1	*A. baumannii*	Rep_3	WP_000818857	pOIFC189-111	*A. baumannii*
Rep_3	WP_000743064.1	pAB3	*A. baumannii*	Rep_3	WP_001205343	pPKAB07	*A. baumannii*
Rep_3	WP_000845851.1	pCMC8300(a)	*A. baumannii*	Rep_3	CAP02936.1	p1ABSDF	*A. baumannii*
Rep_3	WP_005804946.1	pCMC8300(b)	*A. baumannii*	Rep_3	CAP02944.1	p2ABSDF(a)	*A. baumannii*
Rep_3	YP_213946.1	pMAC	*A. baumannii*	Rep_3	CAP02966	p2ABSDF(b)	*A. baumannii*
Rep_3	YP_001736296.1	pABIR	*A. baumannii*	Rep_3	CAP02976	p3ABSDF(a)	*A. baumannii*
Rep_3	YP_002967453.1	pABVA01	*A. baumannii*	Rep_3	CAP02983.1	p3ABSDF(b)	*A. baumannii*
Rep_3	YP_003293957	pMMCU2	*A. baumannii*	Rep_3	CAP02992	p3ABSDF(c)	*A. baumannii*
Rep_3	YP_006960403	pMMCU3	*A. baumannii*	Rep_3	ADX94286	p1ABTCDC0715	*A. baumannii*
Rep_3	YP_006961790.1	pMMD	*A. baumannii*	Rep_3	WP_000095317	pABUH5-114	*A. baumannii*
Rep_3	YP_006964864	pAB120	*A. baumannii*	Rep_3	WP_032021082.1	pABUH2a-5.6	*A. baumannii*
Rep_3	YP_008090880.1	pAB-NCGM253	*A. baumannii*	Rep_3	WP_005804946.1	pABUH3b-7.8	*A. baumannii*
Rep_3	YP_008658574	p1ABIBUN	*A. baumannii*	Rep_3	WP_000818857	pABUH4-111	*A. baumannii*
Rep_3	YP_009066530	pA85-2cloneGC1	*A. baumannii*	Rep_3	WP_001208778.1	pABUH6b-10	*A. baumannii*
Rep_3	YP_009066626	pAB-G7-1	*A. baumannii*	Rep_3	WP_005804946.1	pABUH3a-8.2	*A. baumannii*
Rep_3	YP_009070331.1	AbATCC329	*A. baumannii*	Rep_3	WP_004282236.1	pABUH2b-5.4	*A. baumannii*
Rep_3	WP_001205343	pD36-3	*A. baumannii*	Rep_3	WP_001205343.1	pABUH6a-8.8	*A. baumannii*
Rep_3	WP_000140303	pD36-4(a)	*A. baumannii*	Rep_3	WP_000818856	ZW85p2	*A. baumannii*
Rep_3	WP_000786839	pD36-4(b)	*A. baumannii*	Rep_3	YP_003090213.1	pMMCU1	*A. calcoaceticus*
Rep_3	WP_002124966	II(CIP70.10)	*A. baumannii*	Rep_3	WP_000064928.1	pXM2	*A. calcoaceticus*
Rep_3	WP_002124966	II(R2091)	*A. baumannii*	Rep_3	WP_049068997.1	pXBB1-9	*A. johnsonii*
Rep_3	WP_001205343	p1ABST78	*A. baumannii*	Rep_3	WP_058952620.1	pXBB1-1	*A. johnsonii*
Rep_3	WP_001205343	p2ABST2(a)	*A. baumannii*	Rep_3	WP_058952625	pXBB1-2	*A. johnsonii*
Rep_3	WP_000845976.1	p2ABST2(b)	*A. baumannii*	Rep_3	WP_058952636	pXBB1-3	*A. johnsonii*
Rep_3	WP_001205343	pAB0057	*A. baumannii*	Rep_3	WP_001205341	pXBB1-4	*A. johnsonii*
Rep_3	WP_001205343	pAB5075	*A. baumannii*	Rep_3	WP_006582659.1	pXBB1-5	*A. johnsonii*
Rep_3	AHX30527	pAC12	*A. baumannii*	Rep_3	WP_058952649.1	pXBB1-6	*A. johnsonii*
Rep_3	AHX67213	pAC30a	*A. baumannii*	Rep_3	WP_004637173.1	pXBB1-8	*A. johnsonii*
Rep_3	WP_001205343	pACICU1(a)	*A. baumannii*	Rep_3	WP_000064928	p6411-89.111	*A. nosocomialis*

Table S4 (Continue)

Protein	GenBank Accesion N°	Plasmid	Genus/Species
Rep_3	WP_000845976	pACICU1(b)	*A. baumannii*
Rep_3	ABO13860.1	pAB1	*A. baumannii*
Rep_3	ABO13861	pAB2	*A. baumannii*
Rep_3	WP_001031297	p1ABAYE	*A. baumannii*
Rep_3	WP_001205343	p2ABAYE	*A. baumannii*
Rep_3	WP_000064928	p3ABAYE	*A. baumannii*
Rep_3	WP_000534216	p2BJAB07104	*A. baumannii*
Rep_3	WP_001205343	p1BJAB0868	*A. baumannii*
Rep_3	WP_000534216	p3BJAB0868	*A. baumannii*
Rep_3	WP_001205343	pCanadaBC5-8.7	*A. baumannii*
Rep_3	WP_000845851	pD1279779	*A. baumannii*
Rep_3	WP_001208779	pIS123-12	*A. baumannii*
Rep_3	WP_000185725	pIS123-18	*A. baumannii*
Rep_3	WP_000845851	pABLAC1	*A. baumannii*
Rep_3	WP_000818857	pABTJ2	*A. baumannii*
Rep_3	WP_000534216	pMDR-ZJ06	*A. baumannii*
Rep_3	WP_001205343	pMRSN58-8.7	*A. baumannii*
Rep_3	WP_001205343	pMRSN7339-8.7	*A. baumannii*
Rep_3	WP_050049553	p6411-66.409(a)	*A. nosocomialis*
Rep_3	WP_050049564	p6411-66.409(b)	*A. nosocomialis*
Rep_3	YP_009068008	pMS32-3	*A. pittii*
Rep_3	YP_009070446	pMS32-1	*A. pittii*
Rep_3	WP_005407388	pWCA157-7.5	*A. radioresistens*
Rep_3	WP_005108939	pWCA157-71(a)	*A. radioresistens*
Rep_3	WP_004782596.1	pWCA157-71(b)	*A. radioresistens*
Rep_3	WP_005407445	pWCA157-53	*A. radioresistens*
Rep_3	YP_009067182	pM131-8	*Acinetobacter sp.*
Rep_3	YP_009067189	pM131-6	*Acinetobacter sp.*
Rep_3	YP_009067213	pM131-3	*Acinetobacter sp.*
Rep_3	YP_009070345	pM131-5	*Acinetobacter sp.*
Rep_3	YP_009070355	pM131-2	*Acinetobacter sp.*
Rep_3	YP_001661463	pAV1	*A. venetianus*
Rep_3	YP_001661474	pAV2	*A. venetianus*
Rep_3	WP_002046604	pAV3(a)	*A. venetianus*
Rep_3	WP_019383599	pAV3(b)	*A. venetianus*
Rep_3	WP_005155758.1	pAV3(c)	*A. venetianus*

Table S5. Predicted plasmid conjugation proteins present in nosocomial and environmental plasmid sequences.

Replicon / Contig	Protein name	Pfam Name	Pfam Code	GeneBank Accesion	Genus	% Indentity
pIH1	Conjugation protein TraX	TraX	PF05857	WP_032638097.1	*E. cloacae complex*	100
	Conjugation protein TraI*	-	-	WP_069597126.1	*E.cloacae*	99
pIH2	Relaxase TraI	TrwC	PF08751	AEY80231.1	*K. pneumoniae*	100
	Conjugation protein TraJ	TrwB_AAD_bind	PF10412	WP_063131939.1	*E. coli*	99
	TraK	-	-	AEY80233.1	*K. pneumoniae*	100
	TraL	-	-	YP_004765023.1	*Enterobacteriaceae*	99
	TraM	TrbC	PF04956	YP_001121032.1	*E. coli*	100
	TraA	VirB3	PF05101	YP_004765021.1	*E. coli*	100
	TraB (ATPasa Type VirB4)	CagE, TrbE , VirB	PF03135	KTK22616.1	*E. homaechei*	100
	VirB5	T4SS	PF07996	EHC71373.1	*S. entérica*	100
	EeX	-	-	WP_000858958.1.1	*Enterobacteriaceae*	100
	TraD	TrbL	PF04610	WP_014014948.1.1	*Enterobacteriaceae*	100
	TraE	VirB8	PF04335	WP_001208352.1	*Enterobacteriaceae*	100
	TraO	CagX	PF03524	WP_000758230.1	*Enterobacteriaceae*	100
	TraF	TrbI	PF03743	WP_000101920.1	*Enterobacteriaceae*	100
	TraG	T2SSE	PF00437	WP_001076634.1	*Enterobacteriaceae*	100
	Nuc	PLDc 2	PF13091	WP_000715148.1	*Enterobacteriaceae*	100
pIH6	Conjugation protein TrbL	TrbL	PF04610	WP_004918993.1	*A. baumannii*	99
	Conjugation protein TrbJ	-	-	WP_068564382.1	*A. pittii.*	99
	Mobilization protein	-	-	OCY51628.11	*A. pittii.*	70
	Hypothetical protein	MobA MobL	PF03389	WP_004919014.1	*A. junii*	99
pIH7	Mobilization protein (MobL-like)	MobA MobL	PF03389	WP_068913314.1	*Acinetobacter* sp.	99
	Hypothetical protein	-	-	WP_046128218.1	*Acinetobacter* spp.	100
pIH8	MobA/MobL	MobA MobL	PF03389	EFF84546.1	*Acinetobacter* sp.	100
pIH13	Relaxase MobA	Relaxase	PF03432	CTQ57091.1	*A. lwoffii*	100
	Mobilization protein	MobC	PF05713	WP_050041694.1	*A. parvus*	100
pIH16	Nickase	MobA MobL	PF03389	WP_033107832.1	*A. baumannii*	100
	Mobilization protein	-	-	KQD09980.1	*Acinetobacter* sp.	99
C030	Bacterial conjugation TrbI-like protein	TrbI	PF03743	EEY88456.1	*A. lwoffii*	99
	Conjugative transfer protein TrbG	CagX	PF03524	AOB42326.1	*A. baumannii*	99
	Conjugative transfer protein TrbF	VirB8	PF04335	AOB42316.1	*A. baumannii*	99
	P-type conjugative transfer protein TrbL	TrbL	PF04610	WP_004282003.1	*A. lwoffii*	99
	P-type conjugative transfer protein TrbJ	T4SS	PF07996	EEY88461.1	*A. lwoffii*	100
	Conjugative transfer protein TrbE	CagE_TrbE_VirB	PF03135	AOB42330.1	*A. baumannii*	99
	Conjugal transfer protein TrbC	TrbC	PF04956	EEY88465.1	*A. lwoffii*	99
	P-type conjugative transfer ATPase TrbB	T2SSE	PF00437	WP_004282008.1	*A. lwoffii*	100
C038	IncP-type DNA relaxase TraI	Relaxase	PF03432	AOB42347.1	*A. baumannii*	98
	Type IV secretion system protein VirD4	T4SS-DNA_transf	PF02534	AOB42348.1	*A. baumannii*	99
C046	TraO	-	-	ALD82631.1	Uncultured	90
	Conjugal transfer protein	T4SS-DNA_transf	PF02534	WP_050049539.1	*A. nosocomialis*	91
C047	Conjugal transfer protein TrbI	TrbI	PF03743	WP_005407473.1	*A. radioresistens*	91
C048	TraJ	VirB8	PF04335	ALD82637.1	Uncultured	94
	P-type DNA transfer protein VirB5	T4SS	PF07996	WP_004644764.1	*Acinetobacter* spp.	98
	Type IV secretion system protein VirB6	TrbL	PF04610	AQT19072.1	*A. baumannii*	93
C049	VirB4 family type IV secretion/conjugal transfer ATPase	CagE_TrbE_VirB	PF03135	WP_005248723.1	*Acinetobacter* spp.	96
	Conjugal transfer protein TrbC	TrbC	PF04956	WP_004644768.1	*Acinetobacter* spp.	97

Table S5. Predicted plasmid conjugation proteins present in nosocomial and environmental plasmid sequences. (Continue)

Replicon / Contig	Protein name	Pfam Name	Pfam Code	GeneBank Accesion	Genus	% Indentity
C050	TraR	Relaxase	PF03432	WP_015060264.1	*A. gandensis*	89
C057	P-type DNA transfer ATPase VirB11	T2SSE	PF00437	WP_036388323.1	*M. caprae*	56
	TrwE protein	TrbI	PF03743	WP_034457881.1	*B. koehlerae*	40
	P-type conjugative transfer protein VirB9	CagX	PF03524	WP_039580780.1	*Xanthomonas* spp.	41
	Conjugal transfer protein TraJ	VirB8	PF04335	WP_024640566.1	*P. syringae*	33
	VirB6-like conjugal transfer protein	TrbL	PF04610	AGH89257.1	Uncultured	30
	Hypothetical protein	T4SS	PF07996	WP_049351081.1	*Neisseria sp.*	39
	VirB4 family type IV secretion/conjugal transfer ATPase	CagE_TrbE_VirB	PF03135	WP_036388279.1	*M. caprae*	41
	TraA	SLT	PF01464	ALD82644.1	Uncultured	50
C059	Type VI secretion protein	TrwB_AAD_bind	PF10412	WP_039580787.1	*Xanthomonas* spp.	45
	Conjugative relaxase	TrwC	PF08751	WP_029103647.1	*M. caprae*	33
C063	Major pilus subunit of type IV secretion complex, VirB2	TrbC	PF04956	AQT19076.1	*A. baumannii*	100
	VirB4, major ATPase of Type IV secretion system	CagE_TrbE_VirB	PF03135	AQT19074.1	*A. baumannii*	99
C064	TrbL/VirB6 plasmid conjugal transfer family protein	TrbL	PF04610	EXC31256.1	*Acinetobacter* sp.	99
	P-type DNA transfer protein VirB5	T4SS	PF07996	WP_004644764.1	*Acinetobacter* spp.	99
	TraJ	VirB8	PF04335	ALD82637.1	*Uncultured*	97
C065	Conjugal transfer protein TrbI	TrbI	PF03743	WP_071212497.1	*A. baumannii*	98
C066	Conjugal transfer protein	T4SS-DNA_transf	PF02534	WP_050049539.1	*A. nosocomialis*	94
	TraO	-	-	EXC31248.1	*Acinetobacter* sp.	95
	TraR	Relaxase	PF03432	ALD82671.1	*Uncultured*	97
C112	Nickase	-	-	WP_086194821.1	*Acinetobacter* sp.	90
	Nickase (partial)	MobA_MobL	PF03389	KGH51325.1	*A. idrijaensis*	79
C127	Plasmid mobilization protein	MobA_MobL	PF03389	WP_087015088.1	*A. johnsonii*	84
C135	Relaxase/mobilization nuclease domain protein	Relaxase	PF03432	EXA59620.1	*A. baumannii*	91
C159	MobA/MobL family protein	MobA_MobL	PF03389	EEY88407.1	*A. lwoffii*	79
C161	Nickase	MobA_MobL	PF03389	WP_049069318.1	*Acinetobacter* spp.	96
C188	Hypothetical protein	MobA_MobL	PF03389	WP_068550820.1	*A.pittii*	92
C214	Putative mobilization relaxase	-	-	EXF56472.1	*Acinetobacter* sp.	60
C230	Plasmid mobilization protein	MobA_MobL	PF03389	WP_086373859.1	*A.pittii*	97
C231	Conjugal transfer protein TrbL	TrbL	PF04610	WP_058870616.1	*A. johnsonii*	94
C294	TrbL protein	TrbL	PF04610	EPR86267.1	*A. junii*	99
C303	Hypothetical protein	MobA_MobL	PF03389	OCY52826.1	*A.pittii*	99
C360	IncP-type oriT binding protein TraJ	-	-	AOB42346.1	*A. baumannii*	99

Table S6. Strains and Plasmids used in this work

Strains	Relevant properties	References
A118	*A. baumannii.* Nosocomial isolate. Plasmid free.	Ramirez *et al*., 2012
HB101	*E. coli*. pro, leu, thi, lacY, endA, recA, hsdR, hsdM, Rf^R, Sm^R	Boyer & Roulland-Dussoix, 1969
S17-1	*E. coli.* 294 recA, RP4 integrated, Tc^R	Simon *et a*l. 1983
DH5α	*E.coli*. recA, ΔlacU169, ϕ80dlacZΔM15	Bethesda Res. Lab
Plasmids		
pIHX*	Plasmids isolated from multiresistant isolates from hospital environments	This work
phIHY**	putative phage isolated from multiresistant isolates from hospital environments	This work
pK18	Km^R. High copy number cloning vector	Pridmore, 1987
pK18mob	Km^R. Mobilizable, high copy number cloning vector. Suicide in *A. baumannii* background	Schäfer *et al*., 1994
R751	Tmp^R.IncPβ. Self-transmissible	Jobanputra, & Datta, 1974
R388	Tmp^R.IncW. Self-transmissible	Llosa, *et al*., 1991
RN3	Tc^R.IncN. Self-transmissible	Gotz, *et al*., 1996
pMob6	Km^R. Derived from pK18 with pIH6 Dtr module cloned.	This work
pMob7	Km^R. Derived from pK18 with pIH7 Dtr module cloned.	This work

* Replicons were named as pIHX (plasmid Intra-Hopital), where X corresponded to 1, 2, 3, 6, 7, 8, 9, 11, 12, 13, 14, 15, 16, 17, 18 or 19

** Replicons were named as phIHY (phage Intra-Horpital), where Y corresponded to 4, 5 or 10

Table S7. Primers used in this work

Primer Name	Sequence 5'-3'	Reference
p6MobLS-F	TCCACATGCGAGTAAGACCG	This work
p6MobLS-R	ACCAAAGACAACGGCAGTCA	This work
p7MobLS-F	ATCAAAAGTCGCTAAAACCCCC	This work
p7MobLS-R	TTCGTTTAGCAGCAGATGGGT	This work
p7Rep3-F	ACTTTTCACGTTCCACGCCT	This work
p7Rep3-R	ACGAGCGACACAACGAAAGT	This work
p8RepA-F	TGTGGCTGCATCACTAACACT	This work
p8RepA-R	GCGAAGCGAATTCCGAGTTG	This work
p16Rep3-F	GAAGCGAAAACCAAGGGCAA	This work
p16Rep3-R	TGGAATCCCTCAGTGCGTG	This work

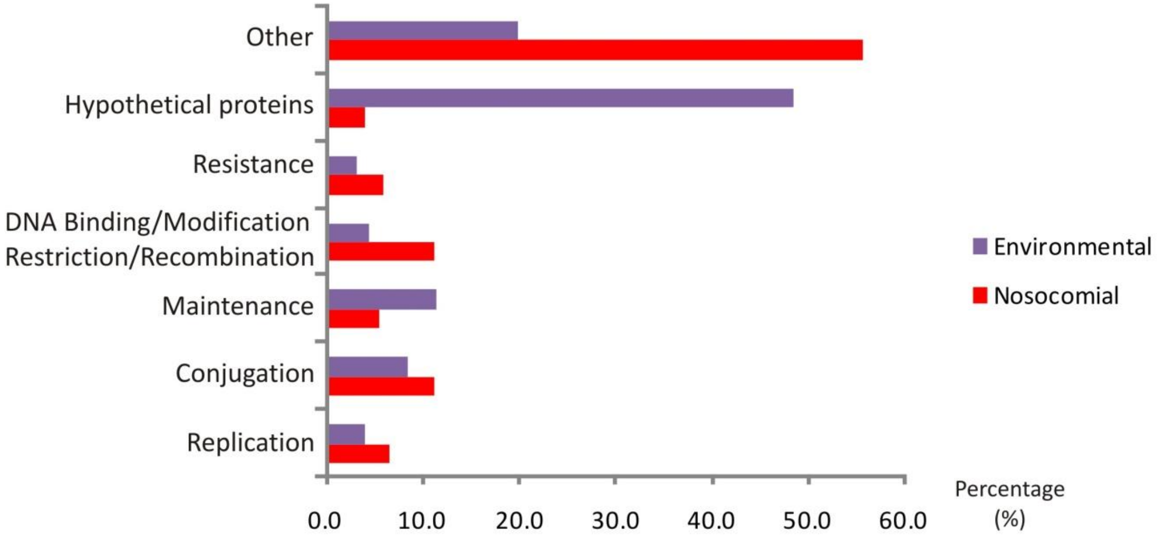

Figure S1. Predicted CDS function distribution.

Percentage distribution of putative plasmids proteins functions from both, environmental and nosocomial collection after *In silico* prediction.

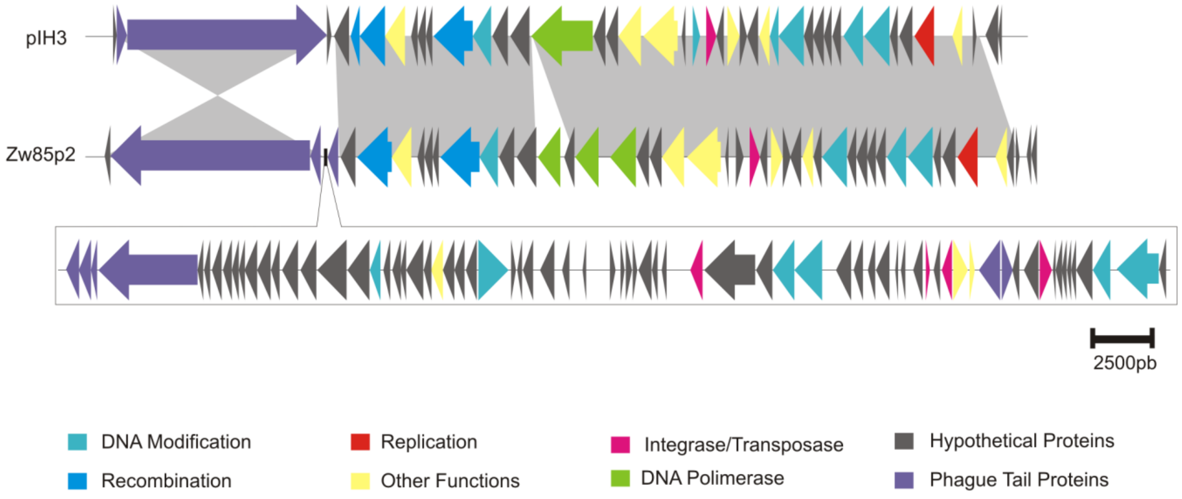

Figure S2. Backbone comparison of plasmid pIH3

Alignment of pIH3 and the closest relative plasmid Zw85p2 (CP006769) is shown. Sequence similarities are highlighted in grey. Inside the white box is represented the 112.8 Kb region absent in pIH3. Functional modules are shown in different colors.

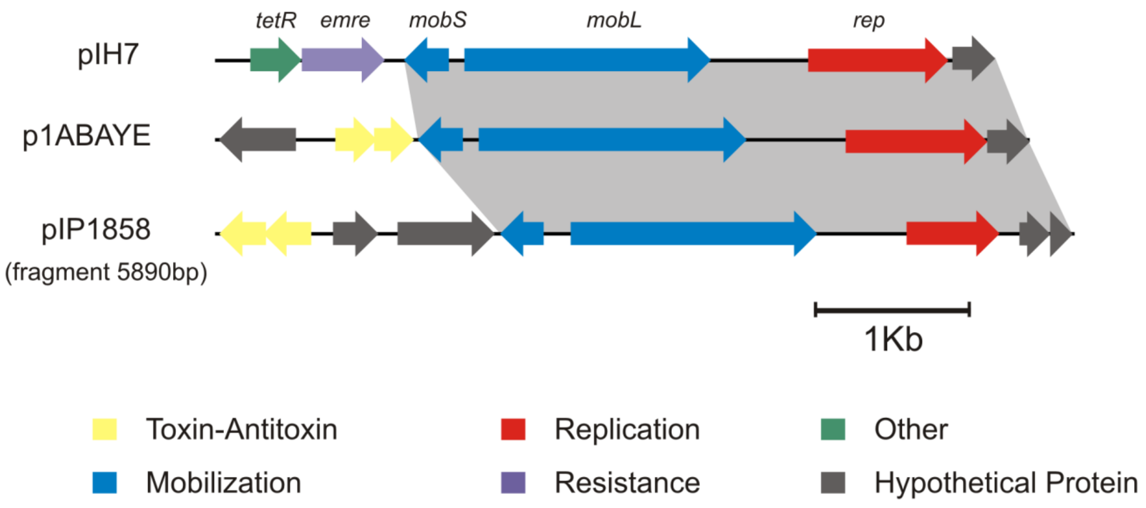

Figure S3. Backbone comparison of pIH7 with other plasmids from database

Alignment of pIH7 with homologue plasmids from database is shown. Sequence similarities are highlighted in grey. Plasmid functional modules are shown in different colors.

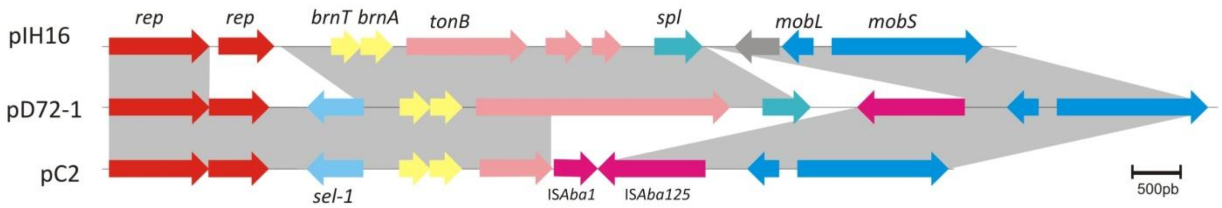

Figure S4. Backbone comparison of pIH16 with other plasmids from database

Alignment of pIH16 with homologue plasmids pD72-1(KM051986) and pC2 (KU549174) from database is shown. Sequence similarities are highlighted in grey. Plasmid functional modules are shown in different colors. (*rep*, replication initiation protein gene; *brnT/brnA*, toxin-antitoxin system; *tonB*, TonB dependent receptor protein gene; *spl*, Septicolysin gene; *mobL/mobS*, conjugation mobilization genes; *sel-1*, Sel-1 domain protein gene)

A

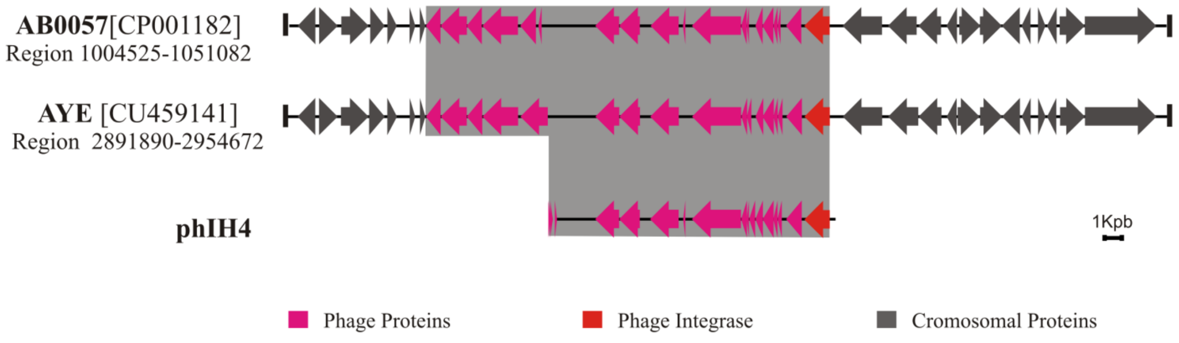

AB0057[CP001182]
Region 1004525-1051082
AYE [CU459141]
Region 2891890-2954672
phIH4
1Kpb
Phage Proteins
Phage Integrase
Cromosomal Proteins

B

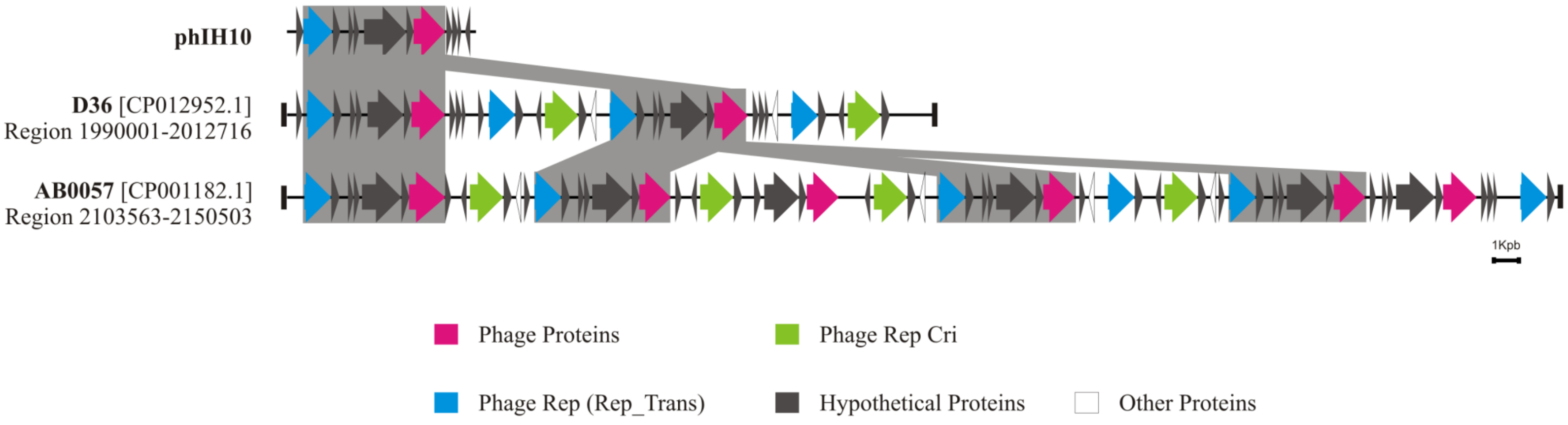

phIH10
D36 [CP012952.1]
Region 1990001-2012716
AB0057 [CP001182.1]
Region 2103563-2150503
1Kpb
Phage Proteins
Phage Rep Cri
Phage Rep (Rep_Trans)
Hypothetical Proteins
Other Proteins

Figure S5. Comparison of phIH4 and phIH10 with *Acinetobacter* spp. chromosome regions.

Sequence similarity of the sequenced replicons with chromosomes regions from other *Acinetobacter* strains in database is shown. A) Alignment of phIH4 with the homologue regions from *A. baumanni* AB0057 and *A. baumannii* AYE strains. Similarities among sequences (highlighted in grey) were confined to all phage proteins. B) Alignment of phIH10 with homologue regions in *A. baumanni* D36 and AB0057 strains. Conserved region among these replicons comprises phage proteins and are found in a repetitive fashion in the mentioned *A.baumanni* chromosomes.

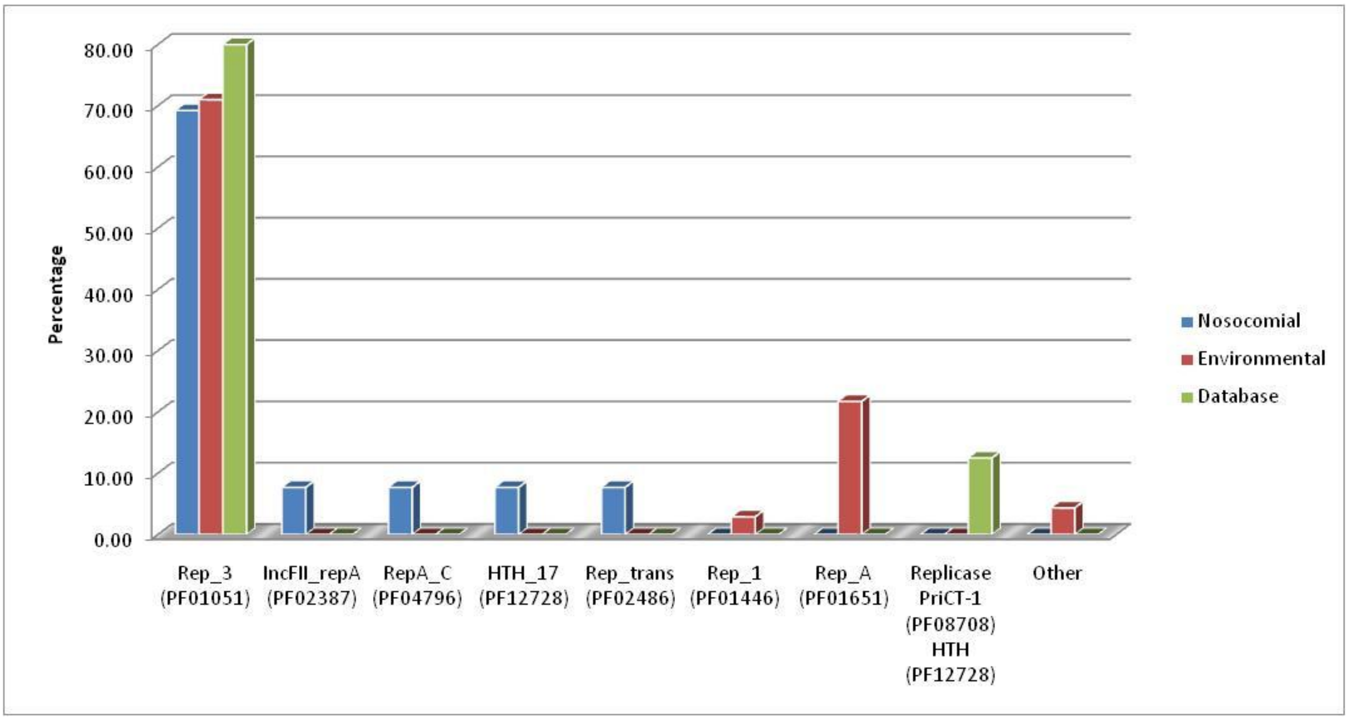

Figure S6. *In silico* replication initiation protein domain distribution

Distribution of Pfam domains found *in silico* in replication initiation protein from the nosocomial, environmental plasmid sequence collections, and *Acinetobacter* spp. plasmids from public database is shown.

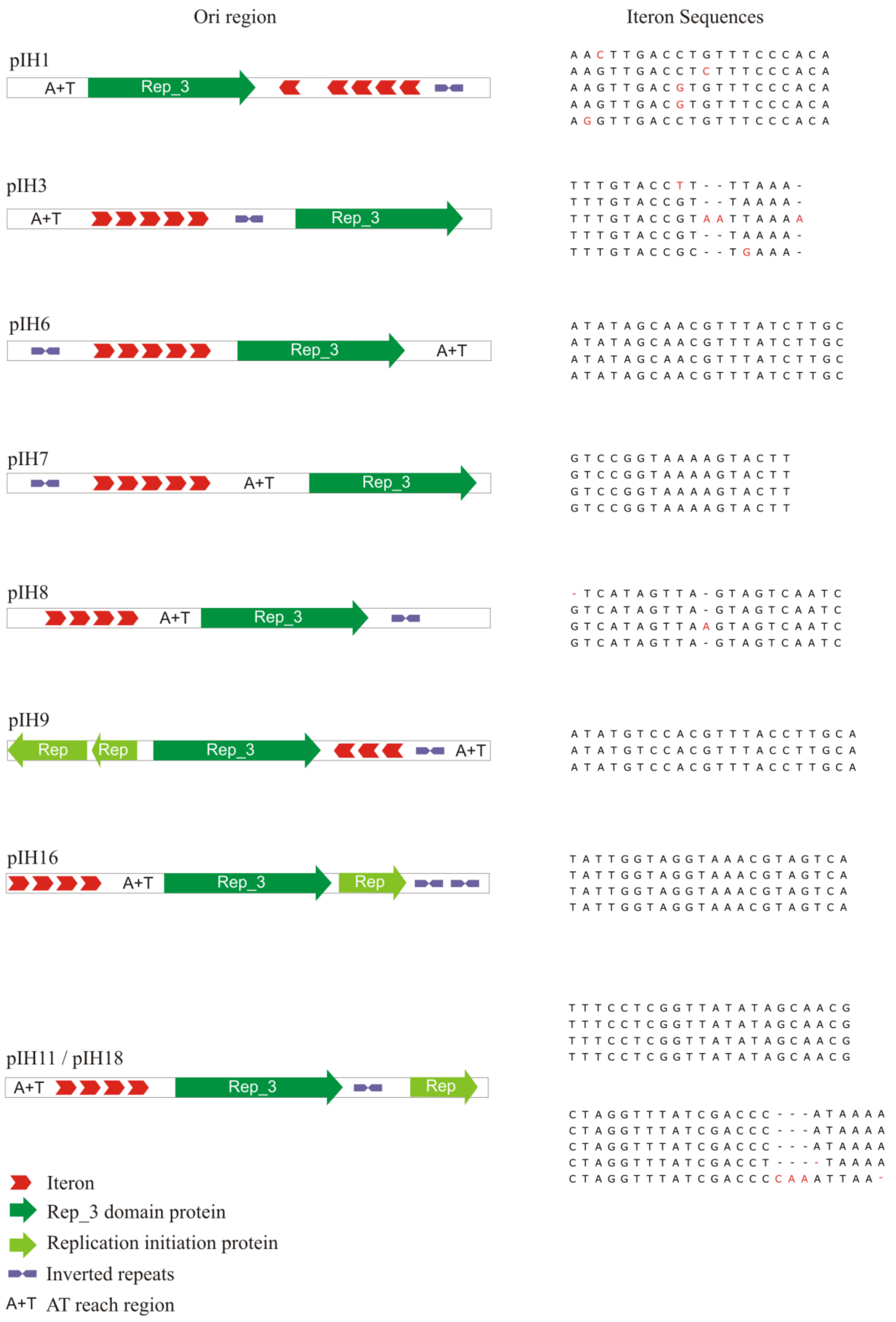

Figure S7. Putative Rep_3 replication origins.

Putative replication origins found *in silico* around Rep_3 domain proteins belonging to nosocomial plasmids sequences is shown. *Ori* features are shows in the graphic in different architectures. Iteron sequences are shown next to the graphic. (Figures are not in scale).

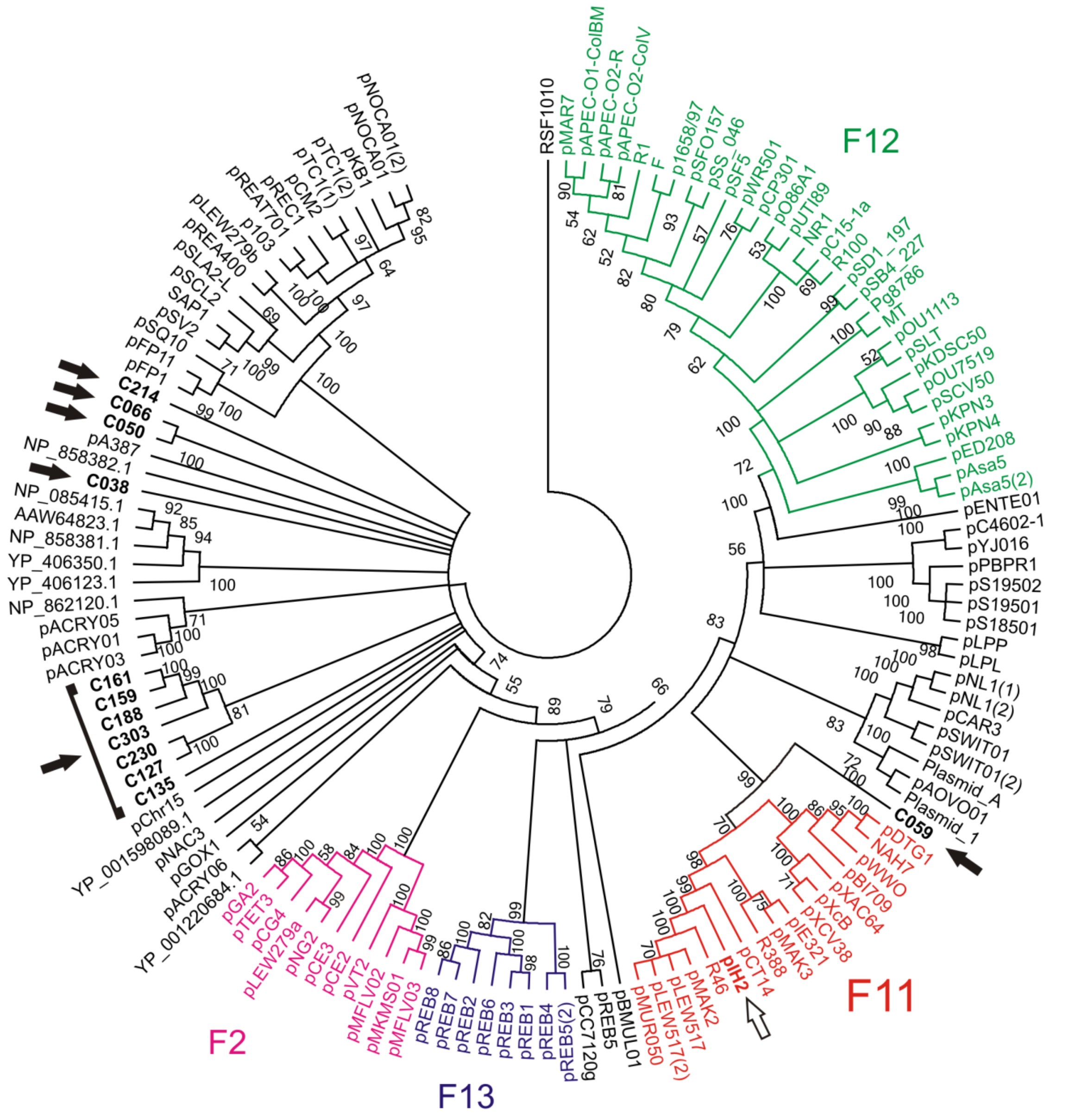

Figure S8. Phylogenetic tree of MOB_F relaxases

Phylogenetic tree of MOB_F relaxasases (Garcillán-Barcia *et al.*, 2009) is shown. Different sub-families are marked in different colors. White arrow points position of pIH2 relaxase from nosocomial plasmid collection. Black arrows point relaxase position of environmental plasmid sequences.

MOB HEN

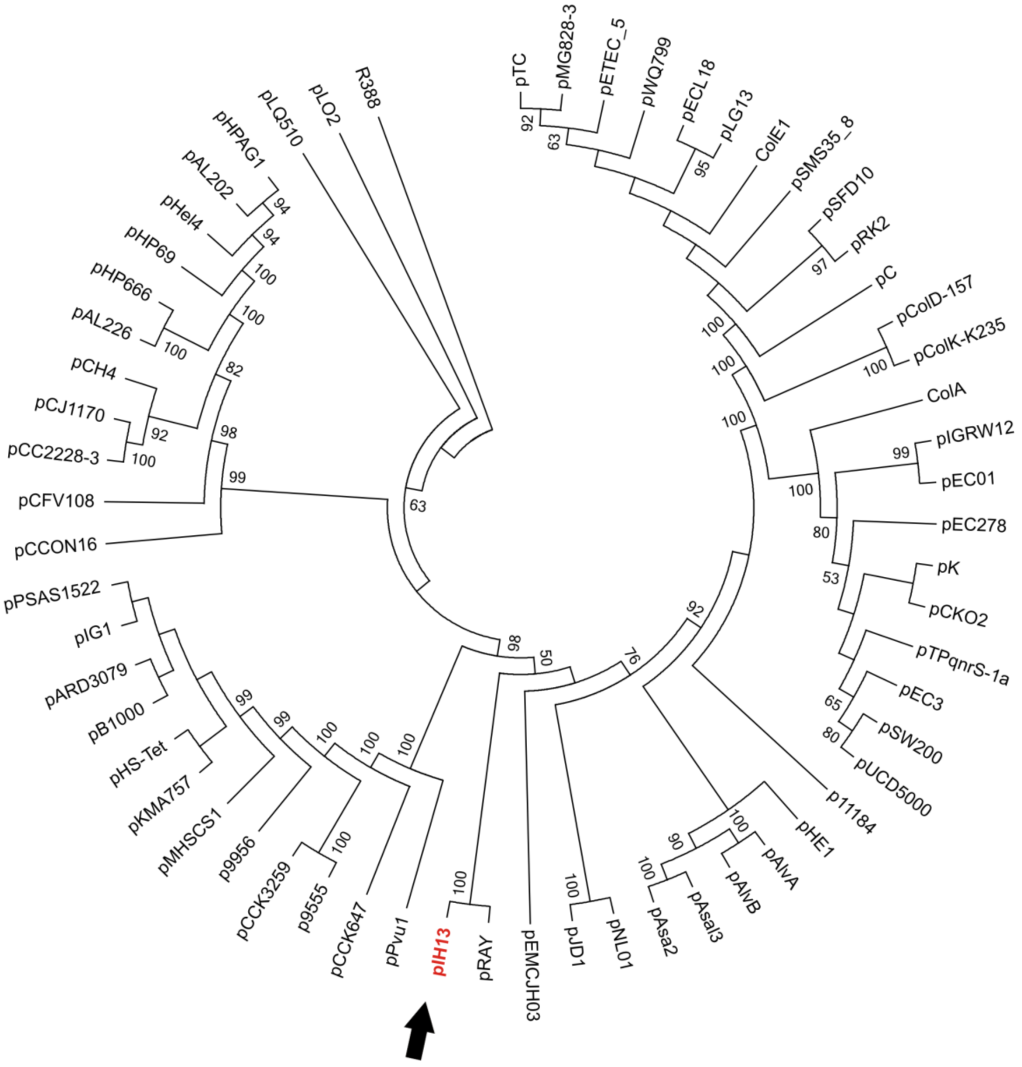

Figure S9.Phylogenetic tree of MOB_{HEN} relaxases

Phylogenetic tree of MOB_{HEN} relaxasases (Garcillán-Barcia *et al.*, 2009) is shown. Black arrow points position of pIH13 relaxase from nosocomial plasmid collection which was classified into MOB_{HEN} family.

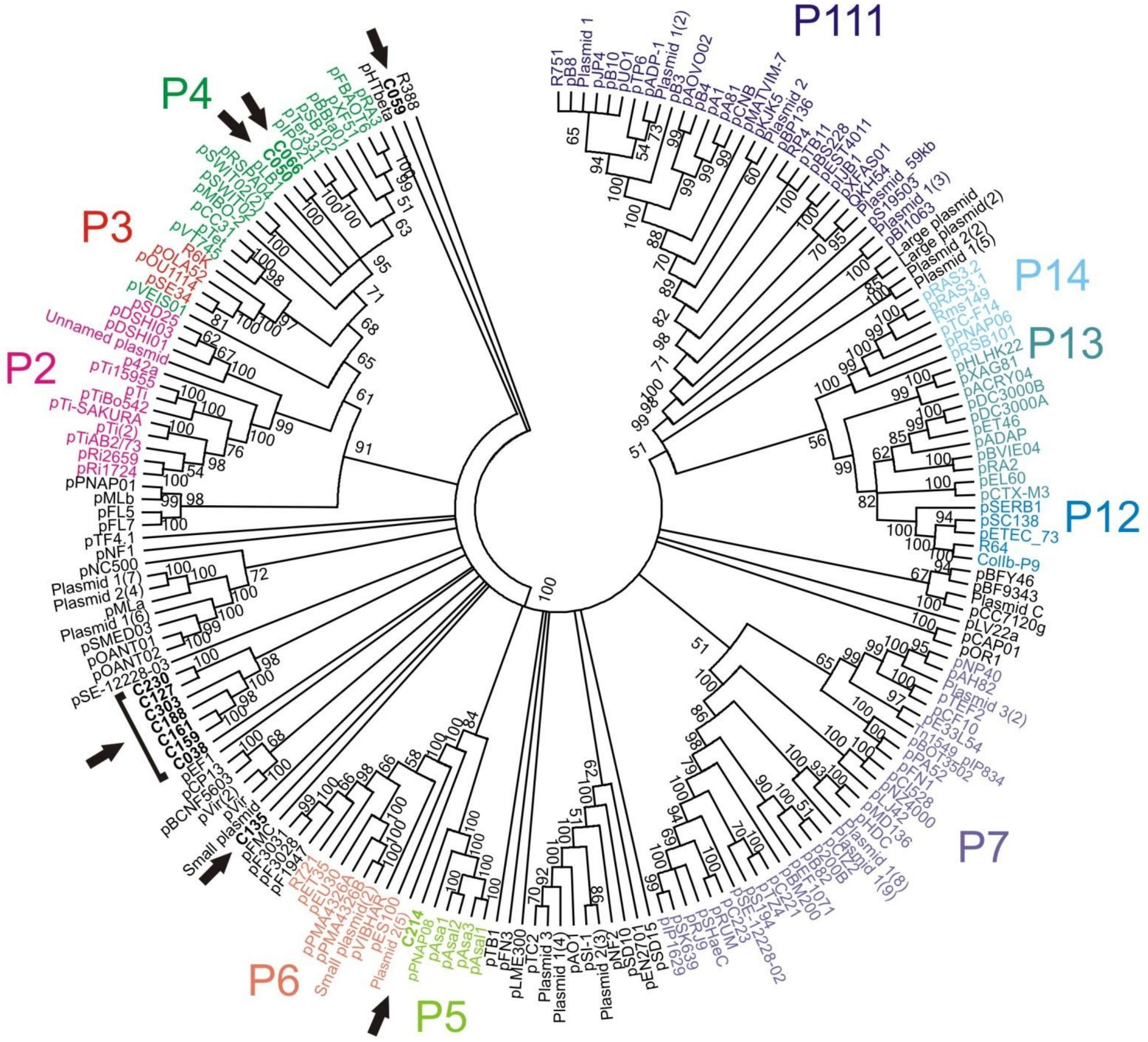

Figure S10. Phylogenetic tree of MOB$_P$ relaxases

Phylogenetic tree of MOB$_P$ relaxasases (Garcillán-Barcia *et al*., 2009) is shown. Black arrows point relaxase position of environmental plasmid sequences.

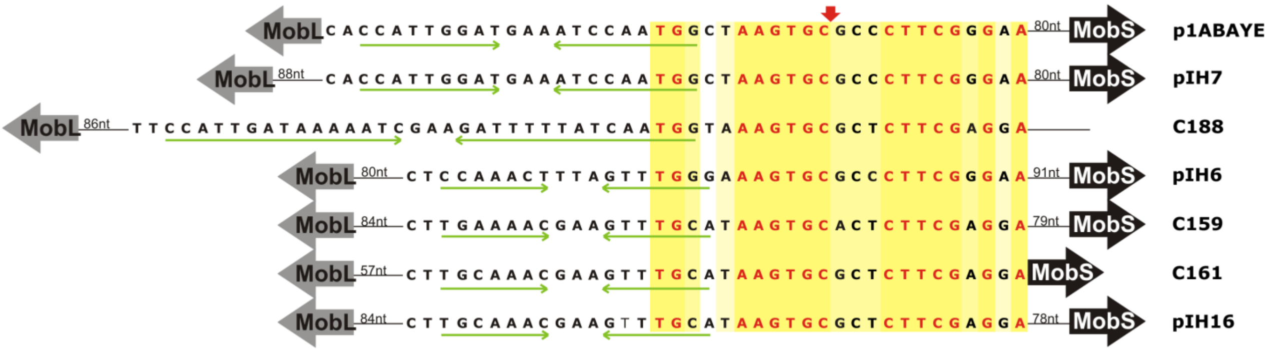

Figure S11. Predicted *oriTs* of MOB$_{\mathrm{QAci}}$ plasmids

Alignment of predicted *oriTs* of MOB$_{\mathrm{QAci}}$ plasmids is shown. Invariant nucleotides are shown in red over yellow. Green arrow represents the inverted repeats of the putative hairpin structure. Red arrow points predicted nick site. Black and grey arrows represent the CDS corresponding to MobS and MobL respectively. Numbers represent nucleotide distance to predicted CDS. Putative *oriT* sites of plasmid p1ABAYE and contig161were (C161) found on the 5' end of MobL and MobS CDS respectively. Prediction of *oriT* regions was done by sequence and structure comparison of *MOBQ$_{Aci}$* plasmid with the previously studied plasmid pTF1 (Drolet *et al*., 1990-1992).
